# Distinct brain and lung endothelial miRNA/mRNA profiles after exposure to *Plasmodium falciparum-*infected red blood cells

**DOI:** 10.1016/j.isci.2024.111265

**Published:** 2024-10-28

**Authors:** Nahla Galal Metwally, Maria del Pilar Martinez Tauler, Hanifeh Torabi, Johannes Allweier, Sara Mohamed, Maryeva Bessemoulin, Philip Bouws, Fatima Alshikh, Yifan Wu, Milad Temori, Tabea Schell, Maximillian Rakotonirinalalao, Barbara Honecker, Katharina Höhn, Thomas Jacobs, Holger Heine, Iris Bruchhaus

**Affiliations:** 1 Research Group Host-Parasite Interaction, Hamburg, Germany; 2 Research Group Molecular Infection Immunology, Hamburg, Germany; 3 Cellular Parasitology Department, Hamburg, Germany; 4 Research Group Protozoa Immunology, Bernhard Nocht Institute for Tropical Medicine, Hamburg, Germany; 5 University of Strasbourg, Strasbourg, France; 6 Division of Innate Immunity, Research Center Borstel, Leibniz Lung Center (Airway Research Center North (ARCN), German Centre for Lung Research (DZL), Borstel, Germany; 7 Biology Department, University of Hamburg, Hamburg, Germany

**Keywords:** Disease, Biological sciences, Neuroscience, Microbiology parasite

## Abstract

MicroRNAs (miRNAs) control 60% of genes expressed in the human body, but their role in malaria pathogenesis is incompletely understood. Here, we demonstrate cell type-specific alterations to the miRNA profiles during the early response to malaria infection in brain and lung endothelial cells (ECs). In brain ECs, incubation with *Plasmodium falciparum*-infected red blood cells in the ring stage (iRBCs) most significantly affected endocytosis-related miRNAs and mRNAs. Contrastingly, in lung ECs, iRBCs altered electron transport chain-related miRNAs and mRNAs. We present a dataset of inherent differences between microRNA profiles in brain and lung ECs and their extracellular vesicles (EVs). We demonstrated that shear stress affected multiple pathways in brain ECs, which were controlled by numerous human miRNAs. Together, these findings indicate that host miRNAs respond to parasite exposure, accompanied by stimulation of downstream signaling pathways within the ECs. Therefore, we consider miRNAs the initial spark for early host-parasite interaction events.

## Highlights

• Shear stress reinforced tight junctions in brain endothelium
• Endocytic pathways were activated in the brain endothelium
• The electron transport chain pathway was activated in lung endothelium
• miRNA candidates might be causing the mRNA alterations within the endothelium

## Graphical abstract

**Figure:**
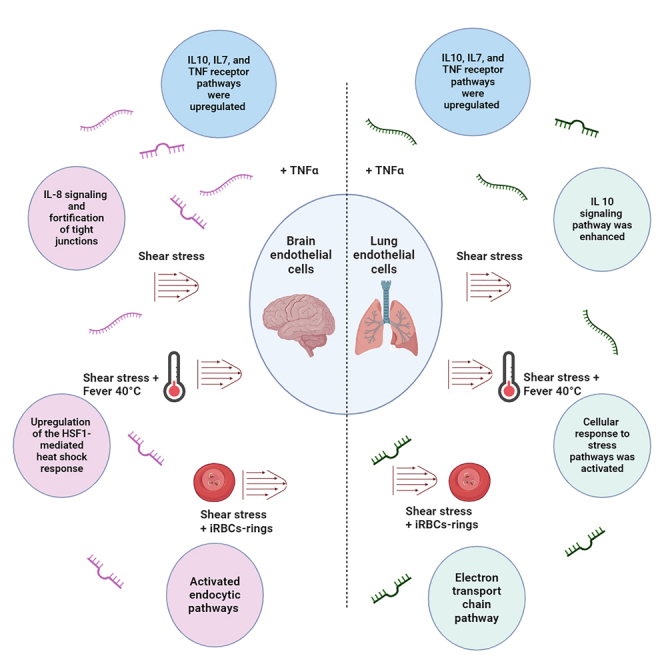

## Introduction

Widespread parasite resistance to artemisinin therapy is hampering malaria eradication programs, and more research into the host response to infection is needed to develop adjuvant therapies that minimize complications.^1^^,^^2^ Malaria deaths reached 608,000 cases in 2022 with 249 million clinical malaria infections. Pregnant women and children under 5 years of age are most likely to develop severe malaria, with children <5 years of age accounting for 67% of all malaria deaths.^3^ Five parasite species cause malaria, and *Plasmodium falciparum* (*P. falciparum*) is the most virulent, responsible for 95% of malaria deaths and 200 million annual clinical infections.^4^ Fatalities occur due to serious complications of malaria. These complications include cerebral malaria (CM), lung injury, renal failure, acidosis, and severe anemia.^5^^,^^6^^,^^7^ The virulence of *P. falciparum* is considered to be due to its ability to modify the erythrocyte surface to adhere and to evade the host immune attack. The major antigenic ligand found to be responsible for the cytoadhesive properties of the infected red blood cells (iRBCs) are members of the *P. falciparum* erythrocyte membrane protein-1 (*Pf*EMP1) family.^8^^,^^9^

The pathogenesis of severe malaria is hotly debated; some researchers suggest that cytoadhesion is the overriding pathogenic mechanism, while others believe that inflammatory processes are more important.

Inflammation during malaria infection occurs due to the release of proinflammatory molecules including interleukin (IL)-1β, IL-6, IL-8, IL-12 (p70), interferon (IFN)-γ, and tumor necrosis factor (TNF), which are released to slow down parasite growth and eliminate the infection. Also, some regulatory cytokines such as transforming growth factor β (TGF-β) and IL-10 maintain the balance between the proinflammatory and anti-inflammatory responses. When this balance is disrupted, the exaggerated proinflammatory response leads to significant complications. The release of the cytokines into circulation is usually associated with fever. High body temperature was reported previously to affect the parasite, the host, and their interaction dynamics.^10^ All these events lead to vascular endothelial dysfunction which is the cause of high mortality rate within malaria patients.^11^^,^^12^^,^^13^

Establishing the causal role of any single mechanism in severe malaria in humans is difficult. Thus, this study aimed to investigate the mechanism of the host-parasite interaction in more detail.

In the human host, RBCs and endothelial cells (ECs) are the primary cells that interact with parasites during *P. falciparum* infection. During this host-parasite interaction, biological material is exchanged. This includes the transfer of human microRNA (miRNA) between iRBCs and other non-infected cells within the human host, including non-infected RBCs, ECs, and immune cells.^14^ Although miRNAs are known to control 60% of the human genes, but their role in malaria complications remains incompletely understood.

### miRNAs and isomiRs

miRNAs are non-coding RNAs approximately 20 nucleotides in length, and are found in mammals, plants, and viruses. According to miRBase version 22, the human genome encodes ∼3,000 true mature miRNAs, of which 1,115 are currently annotated.^15^ An individual miRNA can repress tens to hundreds of genes, and miRNAs regulate nearly every cellular and metabolic process.^16^^,^^17^^,^^18^ Since the discovery of miRNA in 1993, miRNA has been increasingly recognized as an essential post-transcriptional gene regulator.^18^^,^^19^ Different steps of miRNA biogenesis occur in the nucleus and cytoplasm. In the nucleus, the corresponding region of the genome is transcribed by RNA polymerase II to produce primary miRNA (pri-miRNA) >200 nucleotides in length. The pri-miRNA is cropped into precursor miRNA (pre-miRNA) of 70 nucleotides in length. The pre-miRNA is then transported into the cytoplasm. In the cytoplasm, the pre-miRNA is processed into mature miRNA, which targets mRNA for repression or degradation.^20^^,^^21^ It was previously thought that miRNAs only negatively regulate target mRNA, but recent observations suggest that miRNAs can both repress and stimulate gene expression depending on various factors such as cellular conditions, sequences, and cofactors.^22^

The discovery of miRNA isoforms, or isomiRs, was facilitated by high-throughput technologies such as deep RNA sequencing. IsomiRs differ from canonical miRNAs in length and/or sequence and are generated via RNA modifications catalyzed by enzymes such as deaminases and exonucleases. These molecules were initially hypothesized to be RNA-sequencing/mapping errors. However, subsequent studies demonstrated that small RNA-sequencing data contained a significantly higher percentage of untemplated nucleotide additions (%NTA) than the expected sequencing error rate calculated using small artificial RNAs. These studies confirm that canonical microRNA sequence modifications are physiological events that occur *in vivo* rather than experimental artifacts, which has been supported by the development of more advanced analysis algorithms. IsomiRs, like their canonical counterparts, have distinct functional roles.^23^^,^^24^^,^^25^^,^^26^

### Vascular endothelium and endothelial shear stress

Approximately 96,000 km of blood vessels contained in the human body are maintained by ECs, a heterogeneous cell type that responds to signals in the microenvironment. ECs line the blood and lymph vessels except for the placenta and form a physical barrier between the blood and tissue.^27^^,^^28^ ECs perform numerous functions that depend on their location and activation status. These include maintaining vascular tone, controlling homeostasis, transporting hormones, and recruiting immune cells. ECs initiate and amplify the inflammatory response to vascular insults and are also known to have tissue-specific heterogeneity.^29^ Vascular ECs sense hemodynamic changes and signals transmitted by the blood and respond by releasing vasoactive substances.^30^ Shear stress affects EC morphology, electrochemical activities, and gene expression. Most importantly, NO release increases in response to shear stress, which is regulated by rapid activation of endothelial nitric oxide synthase 3 (eNOS) and upregulation of eNOS gene expression and transcriptional activation.^31^

### Extracellular vesicles

Extracellular vesicles (EVs) are tiny lipid particles released by cells. They contain proteins, lipids, RNA, and DNA and can transport bioactive molecules. EVs also act as cell-to-cell communication carriers and are involved in antigen presentation and inflammatory activation.^32^^,^^33^

### Vascular cell-pathogen interactions

Jambusaria et al. referred to the genetic signature of ECs as “postal codes” for organ-specific drugs. Identifying the differences in genetic signatures between ECs in different organs provides insight into the molecular underpinnings of EC heterogenicity.^34^ Prior studies have explored the transcriptional landscape of brain ECs, and a smaller number of studies have evaluated the transcriptional profiles of lung ECs, but very few studies have explored the organ-specific heterogenicity of EC miRNA profiles. Lung ECs are in contact with the external environment due to their gas exchange function, and thus must initiate a rapid immune response in the event of infection. Lung ECs also facilitate the entrance of immune cells into the lungs to fight invading bacteria and viruses. Contrastingly, brain ECs prevent toxic molecules from entering the brain and maintain a much tighter barrier structure.^34^ Understanding how ECs of different organs respond to stimuli contributes to drug design for infectious diseases.

In the present study, we designed experiments to investigate the mRNA and miRNA profiles of primary human brain and lung ECs exposed to iRBCs. First, we compared the profiles of both ECs and EVs secreted by ECs to characterize the intrinsic heterogeneity between brain and lung ECs. Second, we exposed brain and lung ECs to authentic shear stress stimulation with similar stress levels to microvessels to create a physiologically relevant model. We subsequently evaluated the cellular response to shear stress and iRBC exposure to identify affected pathways and identify miRNAs that could potentially regulate these pathways.

## Results

### Experimental models

**Figure 1:**
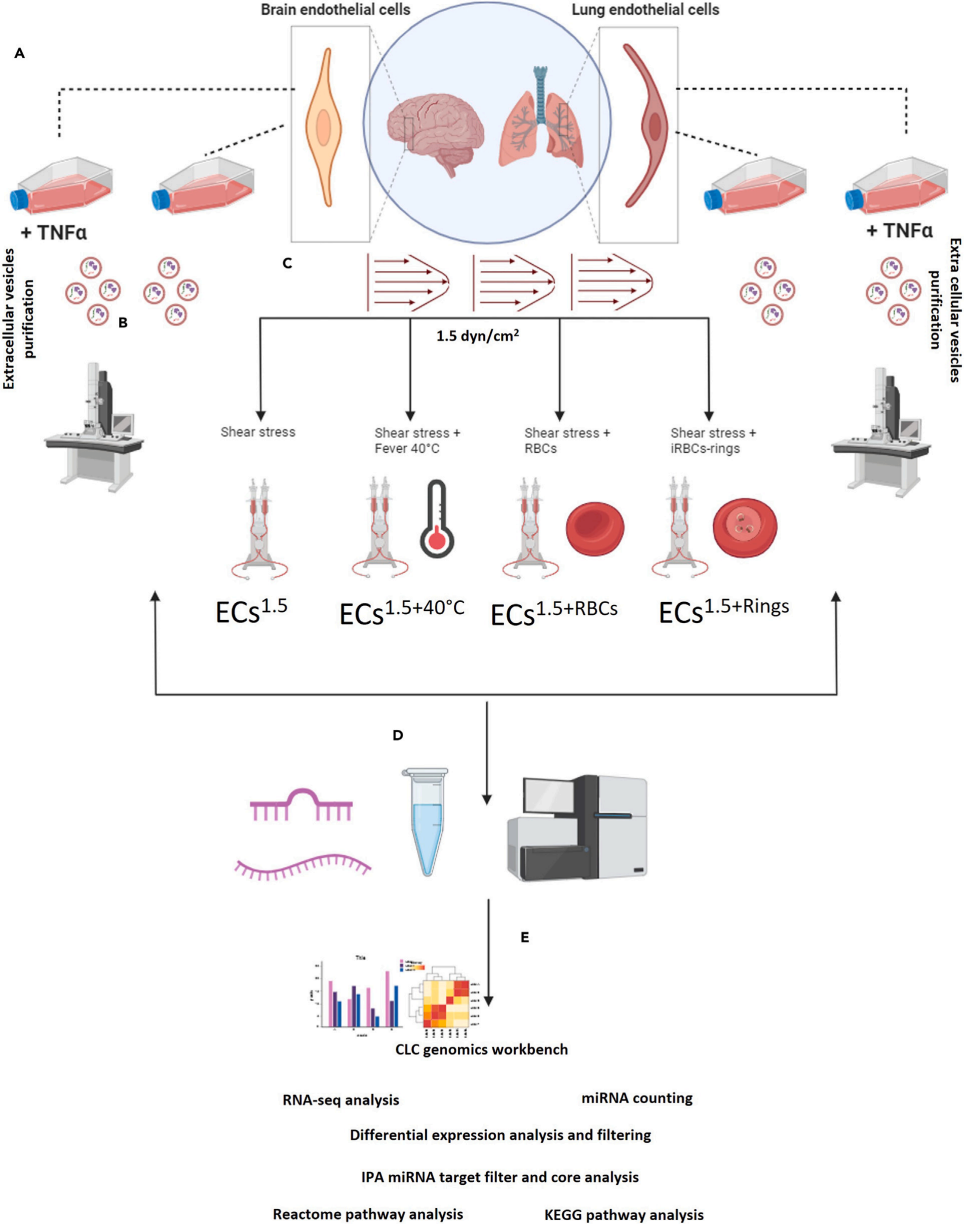
Schematic overview of study workflow (A) Primary brain endothelial cells (ECs) were cultured under static conditions without TNFα (HBMEC^static^) or with TNFα (HBMEC^static+TNF^) as were primary lung ECs (HMVEC-L^static^ or HMVEC-L^static+TNF^). Culture supernatants containing EVs were harvested and sequentially centrifuged to isolate EVs. (B) Transmission electron microscopy was performed to confirm the presence of EVs after purification. (C) A laminar flow system was used to apply shear stress of 1.5 dyne/cm^2^. Stimuli were then added to the fluidic unit: (i) incubation at 40°C (ECs^1.5+40^°C), (ii) non-infected red blood cells (RBCs) (ECs^1.5+RBCs^), and (iii) infected RBCs (iRBCs) (ECs^1.5+Rings^). (D) mRNA/miRNAs were then isolated from ECs and purified EVs and subjected to NGS sequencing. (E) Raw data were extracted and subjected to RNA-seq analysis and miRNA counting using a CLC genomics workbench. Subsequently, differential expression analysis was performed. Finally, miRNA target filtration was performed followed by pathway analysis using Reactome and KEGG pathway analysis.

Cellular miRNA and mRNA sequencing were performed after exposing the ECs to multiple stimuli: (1) shear stress of 1.5 dyne/cm^2^ (ECs^1.5^), (2) shear stress plus fever (40°C) (ECs^1.5+40^°C), (3) shear stress plus uninfected RBCs (ECs^1.5+RBCs^), and (4) shear stress plus ring stage-iRBCs (ECs^1.5+Rings^). The supernatant of static culture was also collected for EV purification and subsequent miRNA isolation and sequencing. Deep NGS sequencing was then conducted, and data were extracted (20 million reads for miRNA and 6–10 million reads for mRNA). The obtained data were analyzed by CLC genomics, Ingenuity Pathway Analysis (IPA), KEGG, and Reactome analyses (Figures 1A–1E). Raw data are uploaded to the NCBI platform (PRJNA1066103).

### Heterogeneity of miRNA and mRNA in brain and lung ECs and secreted EVs

**Figure 2:**
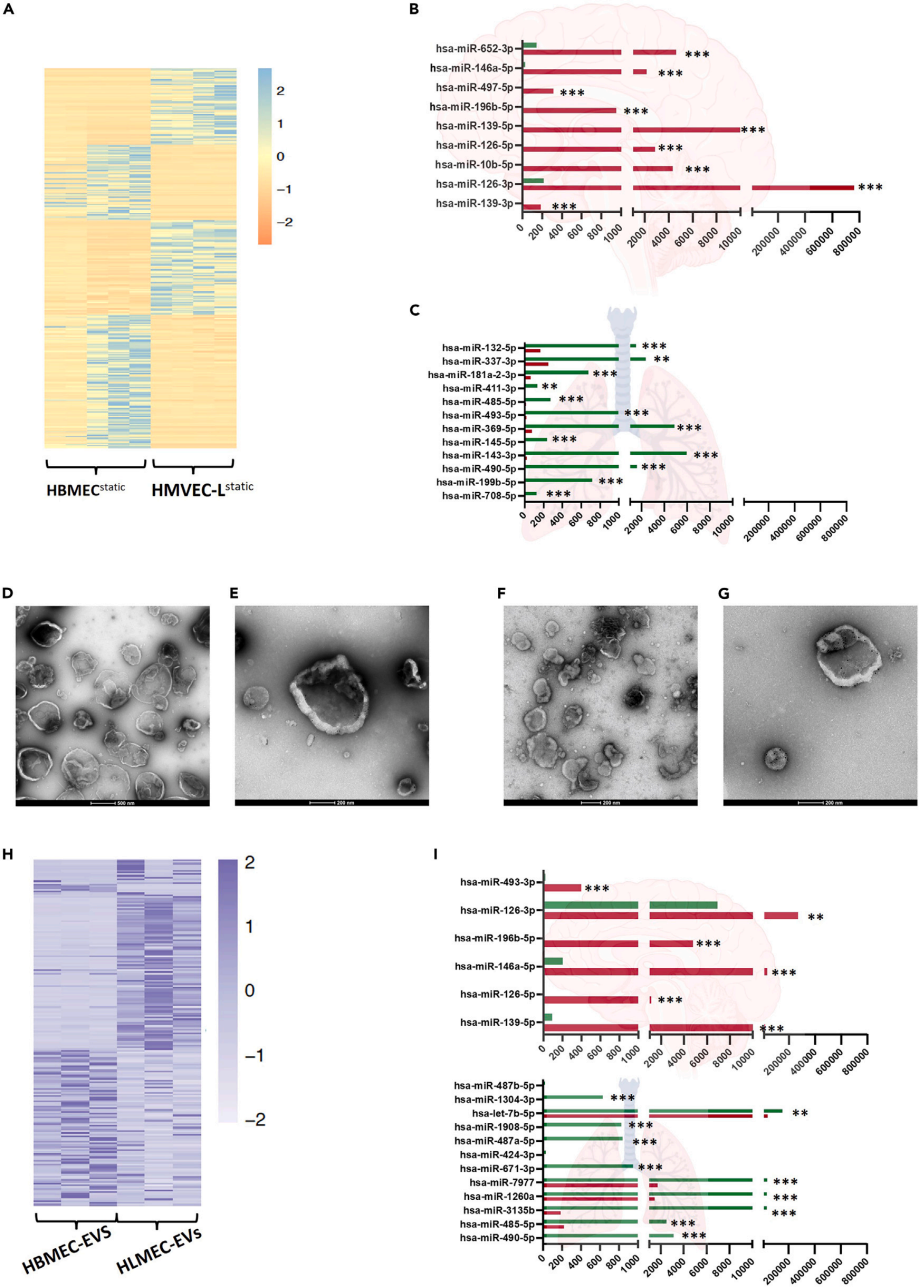
HMVEC-L^static^ versus HBMEC^static^ gene expression analysis (A) Heatmap representing expression levels of the most differentially expressed miRNAs between HBMEC^static^ and HMVEC-L^static^ cells. (B) The nine most highly-expressed miRNAs in HBMEC^static^ cells relative to HMVEC-L^static^ cells. (C) The twelve most highly expressed miRNAs HMVEC-L^static^ relative to HBMEC^static^. (D) Transmission electron microscopy (TEM) images confirming the presence of EVs after purification from HBMEC^static^ culture supernatant. (E) HBMEC-EVs were incubated with anti-CD29 antibody and subjected to immunogold labeling. (F) TEM images confirming the presence of EVs after purification from HMVEC-L^static^ culture supernatant. (G) HMVEC-L-EVs were incubated with anti-CD29 antibody and subjected to immunogold labeling. (H) Heatmap representing expression levels of most differentially expressed miRNAs in HBMEC-EVs and HMVEC-L-EVs. (I) The six most highly-expressed miRNAs in HBMEC EVs and their relative expression, if any, in HMVEC-L-EVs. (J) The twelve most highly-expressed miRNAs in HMVEC-L-EVs and their relative expression, if any, in HBMEC-EVs. Comparisons were performed using the differential expression function in CLC Genomics software V22. Data are represented as the mean of normalized expression values. Statistical significance was calculated based on an FDR-adjusted *p*-value (∗*p* < 0.05, ∗∗*p* < 0.01, ∗∗∗*p* < 0.001 and ∗∗∗∗*p* < 0.0001).

To characterize intrinsic differences between primary brain ECs (HBMECs) and primary lung ECs (HMVEC-L), we performed miRNA/mRNA-seq on both HBMECs and HMVEC-L (Tables S1 and S2). Figure 2A shows a heatmap of miRNAs differentially expressed in lung and brain ECs. Brain ECs exhibited higher expression of 118 mature miRNAs relative to lung ECs, while 152 mature miRNAs were more highly expressed in lung ECs relative to brain ECs (Table S1). Seed miRNAs differentially expressed in lung and brain ECs are blotted in the lower part of the heatmap (Figure 2A). The most significantly altered nine miRNAs with increased brain EC expression level are shown in Figure 2B. The most significantly altered mature miRNAs that were among the most highly expressed in lung ECs from five samples are shown in Figure 2C. Subsequently, we stained brain and lung ECs with the EC markers CD29 and PECAM to confirm their expression on EC surfaces (Figures S1A–S1D).

We then purified and prepared EVs as previously described^35^ for analysis with transmission electron microscopy (TEM). Figure 2D shows TEM images of EVs purified from primary brain ECs (HBMEC-EVs). We then labeled EVs with CD29 (Figure 2E), thus confirming that these EVs originated from ECs. We also purified and labeled EVs from primary lung ECs (HMVEC-L-EVs) (Figures 2F and 2G). Both EV populations retained the normal characteristics of EVs.^36^ Nanoparticle tracking analyses showed EVs populations ranging in size from 105 to 450 nm in case of HBMEC-EVs and 180–240 nm in case of HMVEC-L-EVs (Figures S2A and S2B). Differential miRNA expression analysis revealed 19 mature and 180 seed miRNAs differentially expressed between brain (HBMEC-EVs) and lung (HMVEC-L-EVs) EVs (Figure 2H; Table S3). The six mature miRNAs with significantly higher expression in HBMEC-EVs are plotted in Figure 2I. The twelve mature miRNAs with significantly higher expression in HMVEC-L-EVs are plotted in Figure 2J.

In parallel, we scanned the mRNA profiles for HBMECs and HMVEC-L (Table S2). A total of 3,497 genes were differentially expressed between HBMECs and HMVEC-L. A total of 1,601 genes were exclusively expressed in lung ECs, while 1,896 genes were exclusively expressed in brain ECs.

Reactome pathway analysis revealed three prominent clusters of genes that were highly regulated in each cell type. In brain ECs, the primary clusters were intercellular communication, signal transduction, and the immune response. In lung ECs, the most prominent clusters were extracellular matrix organization, cellular response to stimuli, and hemostasis (Figures S3 and S4).

### TNFα-induced changes in the miRNA and mRNA profiles of brain and lung ECs and secreted EVs

We investigated how the ECs reacted to the potent proinflammatory cytokine TNFα. We stimulated HBMEC^static^ and HMVEC-L^static^ with TNFα (5 μl/5 mL) for 8 h. Table S3 shows the mRNA profiles of HBMEC^static^ and HMVEC-L^static^ cells after stimulation with TNFα compared with those of unstimulated control cells. The expression of 3,414 genes was significantly altered in HBMEC^static+TNFα^ cells compared with their expression in non-stimulated cells. Likewise, the expression of 2,809 genes was altered in HMVEC-L^static+TNFα^ cells compared with those in the unstimulated control cells. Table 1 shows some genes whose expression is known to be altered in ECs after TNFα stimulation. The gene encoding the EC receptor ICAM-1 was highly upregulated in HBMEC^static+TNFα^ and HMVEC-L^static+TNFα^ with fold changes of 17 and 32, respectively. Interestingly, the gene encoding VCAM1 was also highly upregulated in HBMEC^static+TNFα^ and HMVEC-L^static+TNFα^ with fold changes of 27 and 42, respectively. Some genes encoding for CXCL and CCL family proteins were also highly upregulated in both cell types. Table 1 shows that cytokine signaling, TNF receptor, IL-10 signaling, and TRAF6-NFκB-mediated activation pathways were activated in both types of ECs after TNFα stimulation (Figures S5 and S6).

**Table 1:** Highly expressed genes after TNFα stimulation of HBMEC^static+TNFα^ and HMVEC-L^static+TNFα^

mRNA-ID	Fold change in brain ECs after TNF stimulation	Fold change in lung ECs after TNF stimulation
CXCL8	4.7	52
CXCL5	17	13
CXCL1	2	14
CXCL6	4	18
CXCL3	18	8
ICAM1	17	32
VCAM1	27	42
TNFAIP3	16	15

### Effect of shear stress on HBMEC miRNA and mRNA profiles

**Figure 3:**
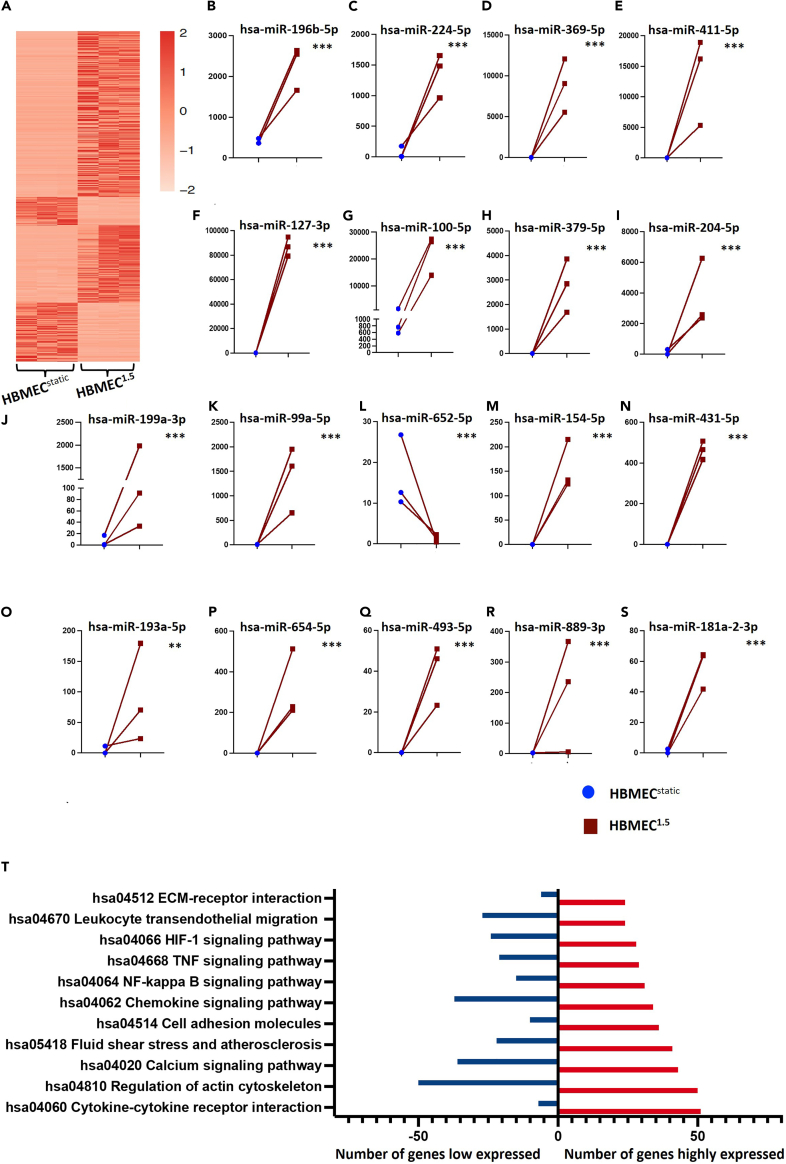
Effects of shear stress on HMBEC miRNA profiles (A) Heatmap representing expression levels of the most differentially expressed miRNAs between HBMEC^1.5^ and HBMEC^static^. (B–S) The 18 most differentially expressed miRNAs in HBMEC^1.5^ relative to HBMEC^static^. (T) Pathways most affected by shear stress in HBMEC^1.5^ relative to HBMEC^static^. Comparisons were performed using the differential expression function in CLC Genomics software V22. Data are represented as mean of normalized expression values. Statistical significance was calculated based on the FDR-adjusted *p* value (∗*p* < 0.05, ∗∗*p* < 0.01, ∗∗∗*p* < 0.001 and ∗∗∗∗*p* < 0.0001).

The EC response to shear stress involves multiple developmental and physiological vascular processes such as angiogenesis, vascular morphogenesis, vascular remodeling, and vascular tone. To identify miRNAs affected by physiological shear stress (1.5 dyne/cm^2^), we compared miRNA expression between HBMECs exposed to 1.5 dyne/cm^2^ shear stress (HBMEC^1.5^) and HBMECs cultivated under static conditions (HBMEC^static^) (Table S4). Figure 3A shows a heatmap representing differentially expressed mature and seed miRNAs in HBMEC^1.5^ cells relative to HBMEC^static^ cells. The 18 most differentially expressed miRNAs are plotted in Figures 3B–3S. In parallel, mRNA from the same samples was sequenced (Table S5). A total of 6,182 genes were affected by shear stress, with 3,693 genes upregulated and 2,489 genes downregulated. Subsequently, we identified the target genes of differentially expressed miRNAs (Table S6). Respectively, pathway analysis identified that miRNAs targeting eleven major pathways were affected by shear stress, with differential miRNA expression in HBMEC^1.5^ relative to HBMEC^static^ (Figure 3T). The most affected pathways were cytokine-cytokine receptor interaction, actin cytoskeleton, calcium signaling, and cell adhesion molecules.

**Figure 4:**
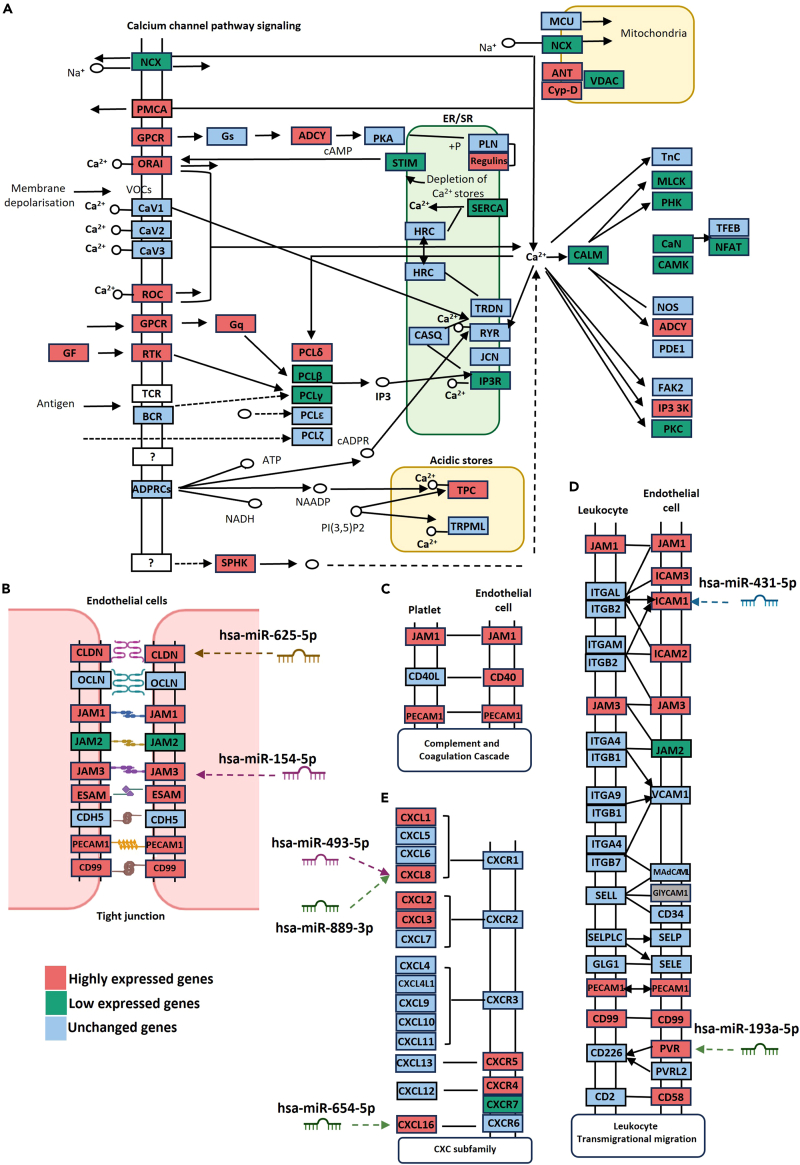
Pathways significantly affected by shear stress in HBMECs (A) Effects of physiological shear stress (1.5 dyne/cm^2^) on calcium channel pathway signaling, as identified by mRNA-sequencing. (B) Effects of shear stress on genes encoding tight junction components. (C) Effect of shear stress on complement and coagulation cascade pathways. (D) Effects of shear stress on leukocyte transmigration signaling. (E) Effect of shear stress on CXC subfamily signaling.

Figure 4A shows which components of the calcium channel pathway were upregulated by shear stress (red), downregulated by shear stress (green), and unchanged (blue), as identified by mRNA-sequencing. This was consistent with prior reports of mechanosensitive calcium-permeable channels in ECs in response to shear stress.^37^^,^^38^^,^^39^^,^^40^^,^^41^^,^^42^^,^^43^

Subsequently, we determined which miRNA candidates within ECs could affect differentially regulated pathways. We identified 33 candidate miRNAs that target genes in calcium channel pathways (Table 2). ECs have a low resting intracellular calcium concentration, which is maintained by plasma membrane calcium-ATPase (PMCA) and sarco/endoplasmic reticulum Ca2^+−^ATPase (SERCA) channels. PMCAs are localized to EC plasma membranes and SERCAs to endoplasmic reticulum membranes. Both classes of ATPases actively transport calcium ions from the cytoplasm to the extracellular space or ER to maintain low intracellular calcium concentrations. Stimulation of G protein-coupled receptors (GPCRs) activates phospholipase C (PLC), triggering hydrolysis of phosphatidylinositol 4,5-bisphosphate (PIP2) to diacylglycerol (DAG) and inositol triphosphate (IP3). IP3 facilitates calcium entry from the sarcoplasmic reticulum and extracellular space. Store-operated cation channels and receptor-operated cation channels facilitate calcium entry from the extracellular space. During stimulation, calcium influx into ECs facilitates dephosphorylation of nuclear factor of activated T-cells (NFAT) from phospho-NFAT (pNFAT) to NFAT. NFAT then translocates to the nucleus,^37^^,^^38^^,^^39^ activating transcription of *ICAM1*.

**Table 2:** Top significantly regulated miRNA candidates and their target mRNAs within the HBMEC^1.5^

miRNA-ID	Fold change	Target mRNA	mRNA fold change	Context++ score percentile	Predicted relative K_D_
hsa-miR-154-5p	−8,157	JAM3	15,516	99	−4.482
hsa-miR-99a-5p	7,437	CLDN11	174,121	95	−4.377
hsa-miR-625-5p	−8,157	CLDN11	174,121	99	−2.957
hsa-miR-92b-3p	76,008	CLDN11	174,121	99	−5.156
hsa-miR-493-5p	−24,471	CXCL8	431,368	91	N/A
hsa-miR-889-3p	−4,894	CXCL8	431,368	99	−5.047
hsa-miR-654-5p	−12,236	CXCL16	68,367	51	−2.424
hsa-miR-193a-5p	−24,471	PVR	191,583	76	−2.675
hsa-miR-431-5p	−6,118	ICAM1	746,207	94	−4.882
hsa-miR-4448	2,738	ICAM1	746,207	97	N/A
hsa-miR-3187-3p	−24,471	CXCL1	10,404	99	−5.019
hsa-miR-155-5p	2,493	ACTA1	−445,009	99	−5.133
hsa-miR-214-3p	−4,894	ATF2	−3,907	97	−4.449
hsa-miR-3613-5p	−24,471	ATF2	−3,907	98	−5.739
hsa-miR-376c-5p	−24,471	ATF2	−3,907	97	N/A
hsa-miR-625-5p	−8,157	ATP2A1	−20,989	81	−1.954
hsa-miR-214-3p	−4,894	ATP2A2	−3,408	92	−4.295
hsa-miR-329-3p	−12,236	ATP2A2	−3,408	N/A	N/A
hsa-miR-625-5p	−8,157	ATP2B4	167,419	61	−2.140
hsa-miR-654-5p	−12,236	CABIN1	−2,257	96	−3.977
hsa-miR-197-3p	−2,039	CACNG8	11,356	84	−3.025
hsa-miR-454-3p	−24,471	CALM2	−3,380	99	−4.616
hsa-miR-193b-3p	−12,236	CALM2	−3,380	N/A	N/A
hsa-miR-660-5p	−24,471	CALM2	−3,380	N/A	N/A
hsa-miR-204-5p	85,448	CAMK1	−2,832	99	−4.840
hsa-miR-3130-5p	−24,471	CHRNA7	44,331	86	N/A
hsa-miR-485-3p	−12,236	CHRNA7	44,331	85	−3.448
hsa-miR-193b-3p	−12,236	CHRNB1	−8,790	97	−4.273
hsa-miR-455-3p	−3,496	CHRNB1	−8,790	N/A	N/A
hsa-miR-455-3p	−3,496	HDAC2	−2,396	N/A	N/A
hsa-miR-454-3p	−24,471	MAPK1	−2,443	96	−4.184
hsa-miR-493-5p	−24,471	MEF2C	−4,689	77	−3.196
hsa-miR-501-5p	−24,471	MEF2C	−4,689	97	N/A
hsa-miR-99a-5p	7,437	PPP3CA	−10,105	94	−4.828
hsa-miR-376a-3p	−4,894	PRKACB	−5,403	98	−4.086
hsa-miR-496	−8,157	PRKACB	−5,403	N/A	N/A
hsa-miR-654-5p	−12,236	PRKAR2A	2,257	80	−3.724
hsa-miR-455-3p	−3,496	PRKD3	−2,904	N/A	N/A
hsa-miR-149-5p	16,673	RAP1A	−2,549	99	−4.440
hsa-miR-337-3p	1,962	RAP1A	−2,549	99	−4.873
hsa-miR-149-5p	16,673	RAP1B	−3,972	97	−2.479
hsa-miR-92b-3p	76,008	RAP1B	−3,972	97	−4.116
hsa-miR-485-3p	−12,236	RAP2A	2,401	94	−3.390
hsa-miR-487a-5p	−8,157	RAP2A	2,401	95	N/A

Secondly, we investigated the effects of shear stress on tight junction (TJ) components in HBMEC^1.5^. TJs are formed by homophilic interactions between transmembrane proteins and junctional adhesion molecules on neighboring cells. TJs determine the tightness of the endothelial barrier by regulating diffusion of fluids, ions, and small plasma proteins and penetration of cells such as leukocytes, neutrophils, and lymphocytes. We identified that out of nine genes encoding TJ components, six were significantly upregulated by exposure to shear stress, including *CLDN*, *JAM1*, *JAM3*, *ESAM*, *PECAM1*, and *CD99* (Figure 4B). Analysis of differentially expressed miRNAs identified that hsa-miR-625-5p, which targets *ATP2B4*, and hsa-miR-154-5p, which targets *JAM3*, are significantly expressed in brain ECs subjected to shear stress (Tables 2, S4, S5, and S6). Shear stress also affected the complement and coagulation cascade, with upregulation of *JAM1*, *CD40*, and *PECAM1* (Figure 4C).

Figure 4D shows genes involved in the leukocyte transmigration pathway. *ICAM-1* was upregulated by shear stress and is targeted by hsa-miR-431-5p. PVR (Polio virus receptor), which was also upregulated by shear stress, is targeted by hsa-miR-193a-5p (Tables 2, S4, S5, and S6). We then evaluated the CxC subfamily pathway, in which seven genes were upregulated by shear stress in brain ECs, including *CXCL1*, *CXCL8*, *CXCL8*, *CXCL16*, *CXCR5*, and *CXCR4*, while only one gene, *CXCR7*, was downregulated. miRNA target analysis identified that both hsa-miR-493-5p and hsa-miR-889-3p target *CXCL8*, while hsa-654-5p targets *CXCL16* (Figure 4E).

### mRNA and miRNA profiles of HBMECs exposed to iRBCs under physiological shear stress

**Figure 5:**
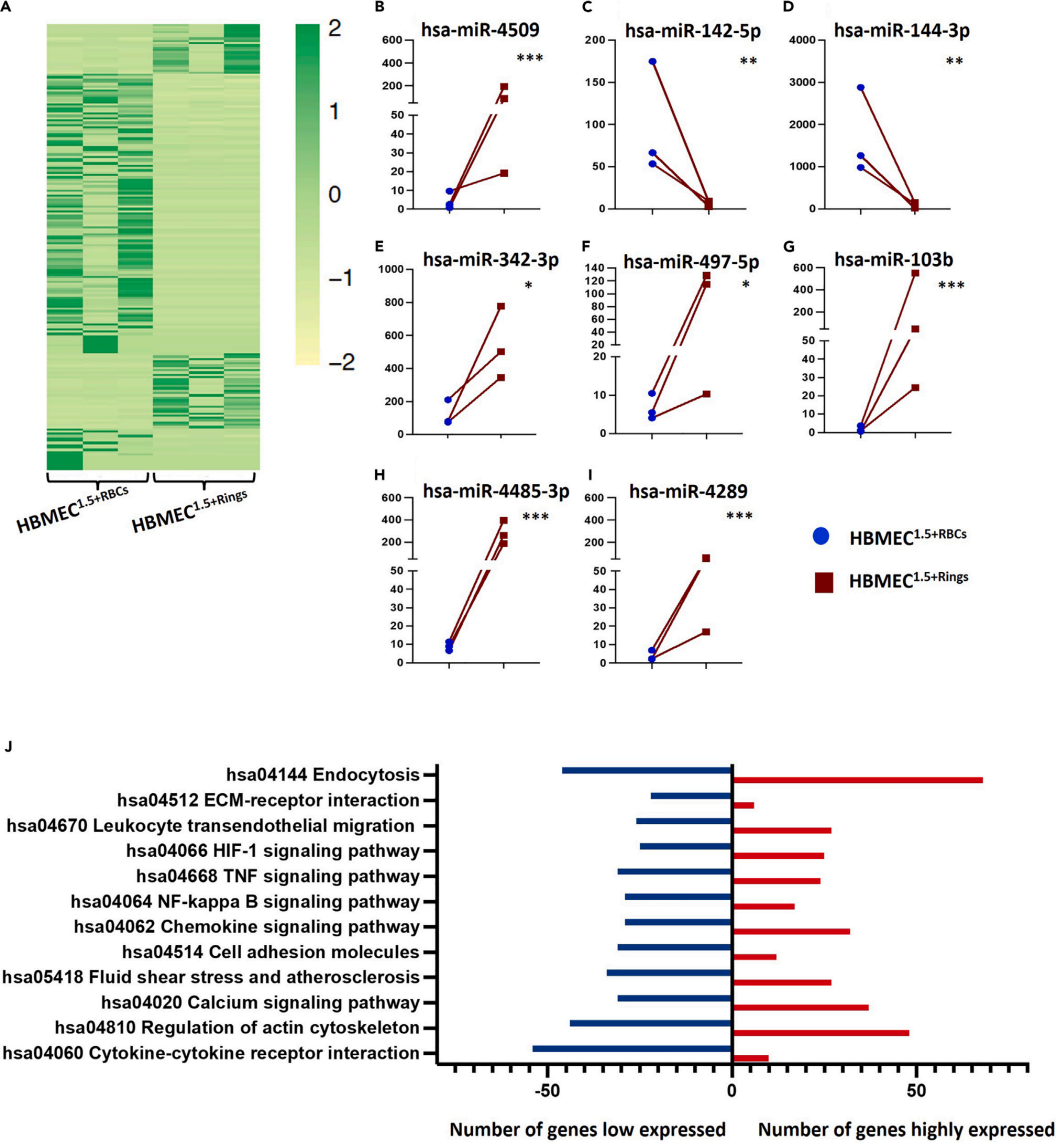
Effects of iRBC exposure on HMBEC miRNA profiles (A) Heatmap showing differential miRNA expression levels between HBMEC^1.5+Rings^ and HBMEC^1.5+RBCs^. (B–I) The eight miRNAs most differentially expressed in HBMEC^1.5+Rings^ relative to HBMEC^1.5+RBCs^. (J) Target pathways most affected by exposure to iRBCs. Comparisons were performed using the differential expression function in CLC Genomics software V22. Data are represented as mean of normalized expression values. Statistical significance was calculated based on an FDR-adjusted *p* value (∗*p* < 0.05, ∗∗*p* < 0.01, ∗∗∗*p* < 0.001 and ∗∗∗∗*p* < 0.0001).

The molecular response of ECs to different stages of *P. falciparum* infection remains incompletely understood. Figure 5A shows a heatmap comparing the miRNA profiles of HBMECs exposed to shear stress and incubated with ring-stage iRBCs (HBMEC^1.5+Rings^) HBMECs exposed to shear stress and co-incubated with non-infected RBCs (HBMEC^1.5+RBCs^) were used. Considering only mature miRNAs, three miRNAs were significantly downregulated, and three miRNAs were upregulated under these conditions. Analysis of miRNA isomers identified 33 miRNA candidates significantly upregulated by exposure to iRBCs and 18 candidate miRNAs significantly downregulated (HBMEC^1.5+Rings^ relative to HBMEC^1.5+RBCs^) (Table S7). Figures 5B–5I shows the top miRNAs that are significantly expressed as a response to the HBMEC^1.5+Rings^. The mRNA target showed that miRNAs regulating the endocytosis, TNF signaling, NF-κB and cytokine-cytokine receptor interaction pathways were affected by iRBC exposure (Figure 5J).

**Figure 6:**
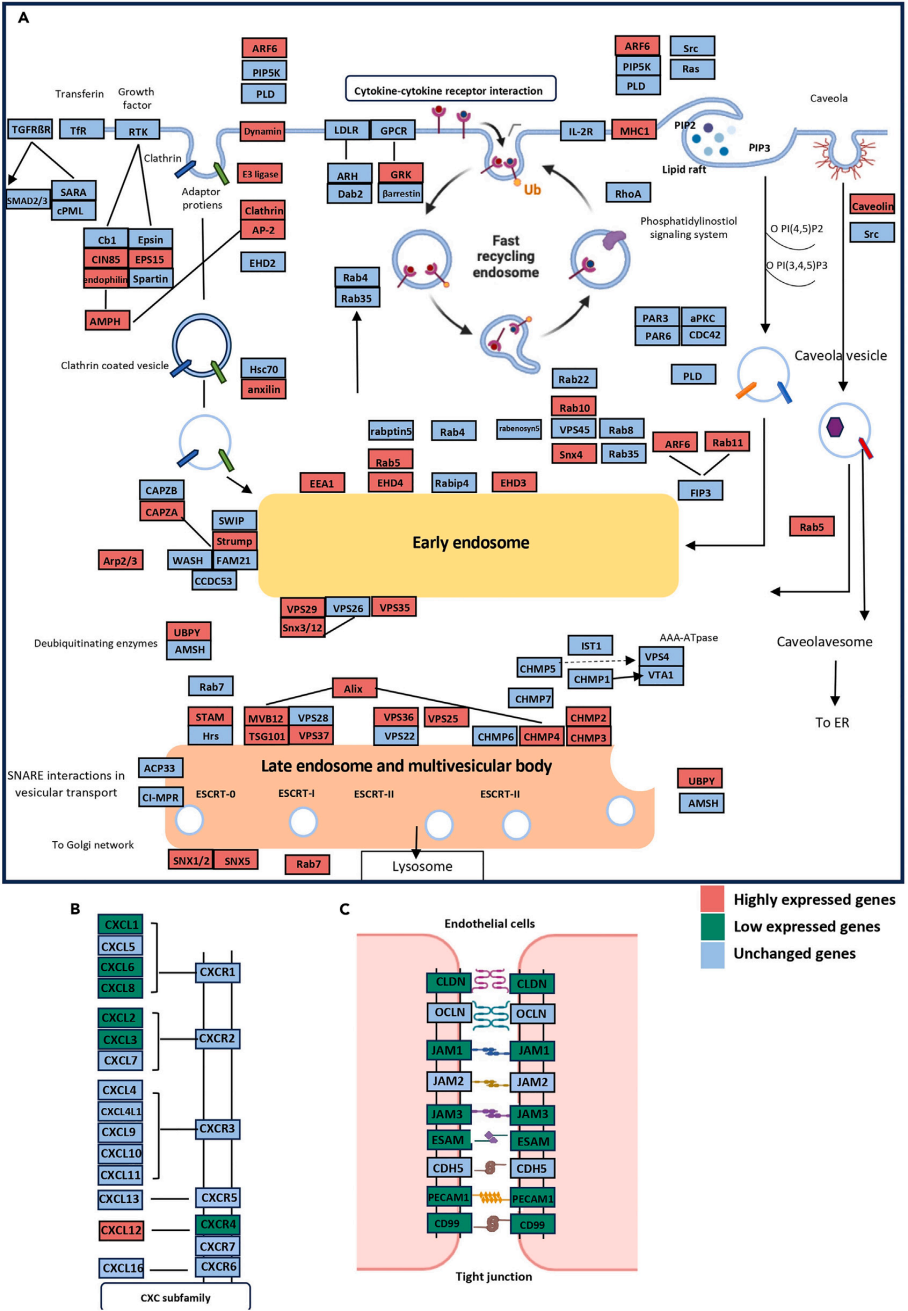
Pathways significantly affected by iRBC exposure in HMBECs (A) The endosomal signaling pathway was affected by co-incubation with ring stage iRBCs and shear stress (1.5 dyne/cm^2^), as demonstrated by mRNA-sequencing. (B) The CXC subfamily signaling pathway was affected by co-incubation with ring stage iRBCs and shear stress 1.5 dyne/cm^2^, with six genes downregulated and one gene upregulated. (C) Co-incubation with ring stage iRBCs affected expression of genes encoding tight unction components in HBMECs, with most genes downregulated in HBMEC^1.5+Rings^ compared with HBMEC^1.5+RBCs^.

Figure 6A shows the endocytosis pathway, with genes upregulated by iRBC exposure indicated in red, downregulated genes indicated in green, and unaffected genes indicated in blue, as demonstrated by mRNA-sequencing analysis. Endocytosis is the cellular process by which substances are taken into cells. The material to be internalized is surrounded by an area of the cell membrane, which then buds off inside the cell to form a vesicle containing the material that has been taken up. Endocytosis is a form of intracellular active substance transport. Endocytic pathways fall into four major categories: receptor-mediated endocytosis (or clathrin-dependent endocytosis), caveolae, pinocytosis, and phagocytosis.

Thirteen genes encoding components of clathrin-dependent endocytosis were upregulated by exposure to iRBCs (Figure 6A). Only two genes involved in clathrin-independent endocytosis (ARF6 and major histocompatibility complex [MHC] I) were upregulated. Genes encoding components of early endosome regulation were also upregulated (10 genes), as were genes encoding components of late endosomes and multivesicular bodies (14 genes) (Figures 6A; Table S7). Immune response pathways, including the CXC subfamily, were also affected in HBMEC^1.5+Rings^ (Figure 6B). Six genes, including *CXCL1*, *CXCL6*, *CXCL8*, *CXCL3*, *CXCL2*, and *CXCR4* were downregulated in HBMEC^1.5+Rings^, while only *CXCL12* was upregulated.

### Effect of shear stress on HMVEC-Ls miRNA and mRNA profiles

We investigated the effects of shear stress on HMVEC-L cells by applying shear stress of 1.5 dyne/cm^2^. A total of 1,174 genes were significantly regulated between cells exposed to shear stress and those unexposed to heat stress (Table S8). One of the main pathways that were stimulated by shear stress was the IL-10 signaling pathway (high expression of *ICAM-1*, *CXCL2*, *IL6*, *CXCL8*, *PTGS2*, and *CXCL3*). IL-4 and -13 signaling pathways were also activated. Conversely, some of the genes in the potassium channel pathway were downregulated (*KCNN2*, *KCNJ8*, *KCND2*, and *KCNK2*) (Table S8). In contrast to brain ECs, TJ genes were not affected by shear stress in the ECs. In the miRNA profile of HMVEC-L cells, only the expressions of 12 isomiRs were significantly altered. The expressions of the following miRNAs were significantly high: hsa-miR-4797-3p, hsa-miR-4670-3p, hsa-miR-6813-3p, has-miR-1286, hsa-miR-6828-5p, hsa-miR-7154-3p, hsa-miR-2467-3p, and hsa-miR-4661-5p. The expression of the following isomiRs was significantly low: hsa-miR-555, hsa-miR-6873-5p, hsa-miR-4725-5p, and hsa-miR-6792-5p.

### mRNA and miRNA profiles of HMVEC-Ls exposed to iRBCs under shear stress

**Figure 7:**
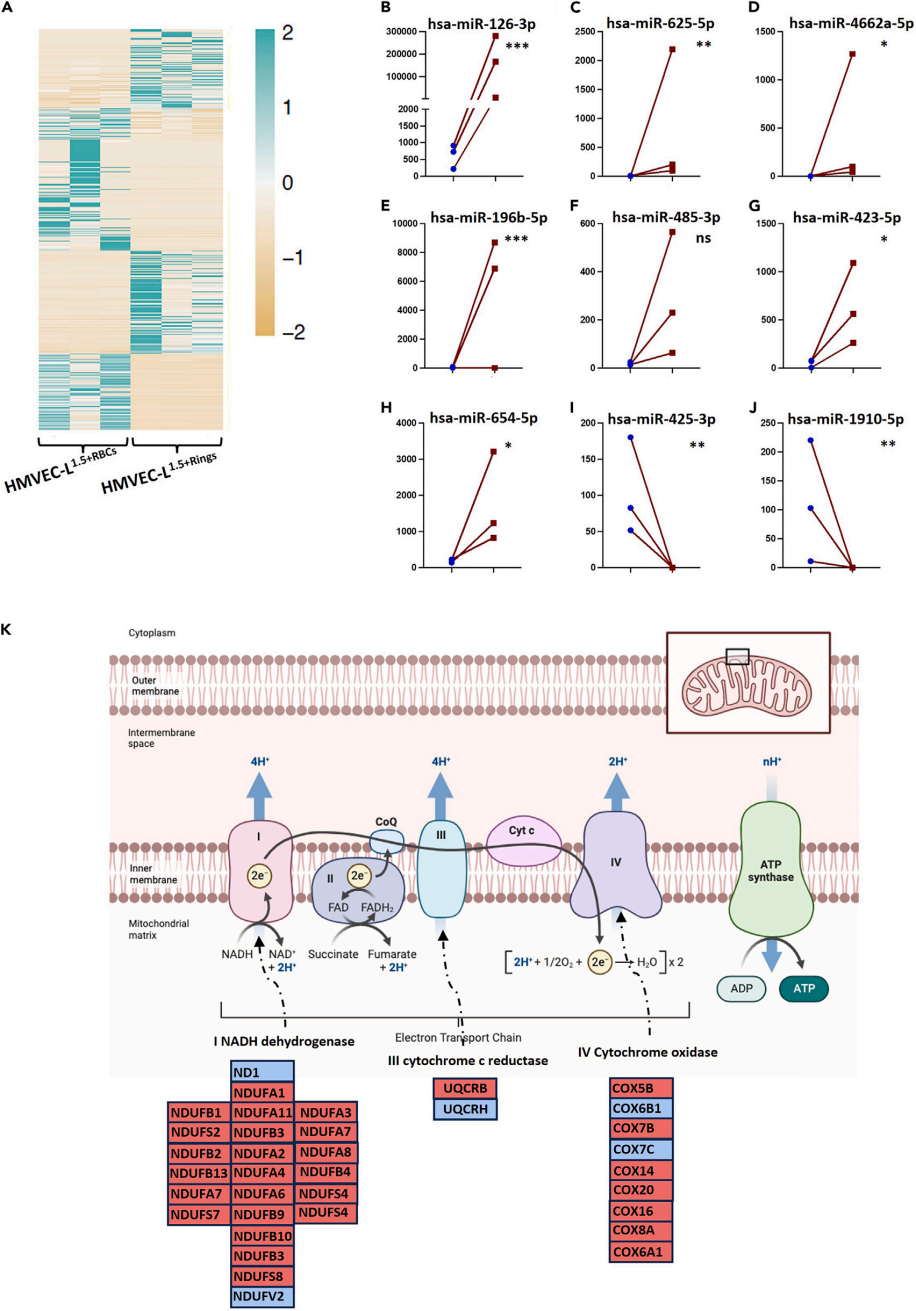
Effects of iRBC exposure on the electron transport chain in HMVEC-Ls (A) Heatmap representing expression levels of the most differentially expressed miRNAs in HMVEC-L^1.5+Rings^ relative to HMVEC-L^1.5+RBCs^. (B–J) The nine miRNAs most significantly affected in HMVEC-L^1.5+Rings^. Comparisons were performed using the differential expression function in CLC Genomics software V22. Data are represented as mean of normalized expression values. Statistical significance was calculated based on an FDR-adjusted *p* value (∗*p* < 0.05, ∗∗*p* < 0.01, ∗∗∗*p* < 0.001 and ∗∗∗∗*p* < 0.0001). (K) Genes encoding components of the electron transport chain were differentially expressed in HMVEC-L^1.5+Rings^ relative to HMVEC-L^1.5+RBCs^, with most genes upregulated.

To determine how lung ECs are affected by the presence of malaria parasites, we incubated lung ECs with infected or uninfected RBCs under shear stress conditions (HMVEC-L^1.5+RBCs^ vs. HMVEC-L^1.5+Rings^). Figure 7A shows a heatmap of miRNAs differentially expressed in HMVEC-L^1.5+Rings^ cells relative to HMVEC-L^1.5+RBCs^ cells. Eighteen mature miRNAs were significantly upregulated and 36 mature miRNAs were significantly downregulated in HMVEC-L^1.5+Rings^ relative to HMVEC-L^1.5+RBCs^. Among isomiRs, 219 miRNAs were differentially expressed in HMVEC-^L1.5+Rings^ relative to HMVEC-L^1.5+RBCs^, with 126 miRNAs upregulated and 93 miRNAS downregulated (Table S9). The miRNAs were then strictly filtered to the nine most significantly affected miRNAs (Figures 7B–7J). We used predictive modeling to identify the target genes of differentially expressed miRNAs (Table S10).

Pathway analysis of mRNA-sequencing data identified that the primary gene set differentially expressed in HMVEC-L^1.5+Rings^ relative to HMVEC-L^1.5+RBCs^ was the ETC (Figure 7K). Out of 35 genes in the ETC pathway, 30 genes were upregulated in HMVEC-L^1.5+Rings^ relative to HMVEC-L^1.5+RBCs^. Table S10 shows miRNA candidates that target genes in the ETC pathway.

### How high temprature (40°C) affects the mRNA and miRNA profiles of HBMEC and HMVEC-L cells under shear stress

Brain ECs reacted to high temperature by upregulating the expression of 97 genes that are involved mainly in the regulation of the HSF1-mediated heat shock response pathway and the cellular response to heat stress pathway (Table S11). In addition, 59 genes were downregulated in response to heat stress. These were involved mainly in the interferon alpha, beta, and gamma signaling pathways. The miRNA profiles of cells exposed to heat stress showed downregulation of about 300 mature miRNAs and the significant high expression of 225 isomiRs (Table S11). IPA miRNA target filter analyses showed that the affected miRNAs and isomiRs target mostly the aforementioned genes within the mRNA profile (Table S11).

Lung ECs reacted to high temperature by upregulating 169 genes involved mainly in the cellular response to stress pathways (Table S12). The miRNA profiles of these cells showed that only eight isomiRs expression levels were affected by heat stress. The expressions of hsa-miR-4442, hsa-miR-6814-5p, hsa-miR-3197, hsa-miR-4501, hsa-miR-491-3p, and hsa-miR-3135a were significantly downregulated. On the other hand, hsa-miR-3606-3p and hsa-miR-520a-5p were significantly upregulated.

## Discussion

EC cell cultivation is usually done under static conditions, which means that the effects of shear stress may go unnoticed. The impact of shear stress on miRNA profiles, especially those in ECs from various organs, has not been studied in detail.

Previous studies characterized the transcriptional profiles of cultured human umbilical vein endothelial cells (HUVEC) exposed to sustained physiological shear stress. The results of these studies suggested that shear stress stimuli alter gene expression in ECs, creating an antioxidant, anti-apoptotic and anti-proliferative environment.^40^

### Brain EC-specific alterations in miRNA/mRNA profiles in response to shear stress

The findings of this study demonstrate that specific miRNAs candidates are highly expressed in brain ECs and their secreted EVs under normal static conditions. These miRNAs were also affected by shear stress. Shear stress of 1.5 dyne/cm^2^, which is similar to that of physiological shear stress in microvessels, significantly altered expression of 18 miRNA candidates significantly, including miR-196b-5p,-224-5p,-369-5p, -411-5p, -127-3p, -100-5p, -379-5p, -204-5p, -199a-3p, -99a-5p, -652-5p,-154-5p, -431-5p, -193-5p, -654-5p,-493-5p, -889-3p, and -181a-2-3p. A prior study reported six mechano-sensitive endothelial miRNAs, including hsa-miR-8060, -4534, -630, -5703, -1587, -1268a, and -4788,197-5p.^41^^,^^42^^,^^43^ However, the present study did not affect these miRNAs by shear stress. This could be because the investigators subjected ECs to higher shear levels (4 dyne/cm^2^ and 100 dyne/cm^2^) than that used in the present study (1.5 dyne/cm^2^), which is like physiological shear stress in microvessels. Consistent with our findings, prior studies have demonstrated that shear stress decreases expression of mechanosensitive miR-181b-5p, which suppresses NLRP3 inflammasome-dependent pyroptosis. Clinical data are also consistent with this notion, revealing that miR-181b acts in combination with lncRNA ANRIL to mediate NF-κB signaling.^44^^,^^45^^,^^46^^,^^47^^,^^48^^,^^49^

We demonstrated that shear stress increased expression of genes encoding TJ components in brain ECs, which are likely targeted by miR-625-5p and -154-5p. These results are consistent with a recent study demonstrating that laminar flow protects vascular endothelial junctions.^50^^,^^51^^,^^52^ We also found that shear stress upregulated CXCL1, 8, 2, 3, and 16 in brain ECs. These genes were likely targeted by hsa-miR-493-5p, -889-3p, and 654-5p, which is consistent with a prior study by Shaik et al. reporting that exposure to shear stress increases EC secretion of CXC chemokines.^50^^,^^51^^,^^52^

### Lung ECs response to physiological shear stress

EC type and function are known to differ between organs,^34^ suggesting that they might respond differentially to shear stress stimuli. In this study, the lungs showed upregulation of the IL-10 signaling pathway in response to shear stress of 1.5 dyn/cm^2^ (high expression of *ICAM-1*, *CXCL2*, *IL6*, *CXCL8*, *PTGS2*, and *CXCL3*). IL-4 and -13 signaling pathways were also upregulated. TJ genes in the lung ECs were not affected by shear stress. In the miRNA profile of HMVEC-L cells, only the expressions of 12 isomiRs were significantly upregulated. The expressions of the following miRNAs were highly upregulated: hsa-miR-4797-3p, hsa-miR-4670-3p, hsa-miR-6813-3p, has-miR-1286, hsa-miR-6828-5p, hsa-miR-7154-3p, hsa-miR-2467-3p, and hsa-miR-4661-5p. On the other hand, the expressions of the following isomiRs were downregulated: hsa-miR-555, hsa-miR-6873-5p, hsa-miR-4725-5p, and hsa-miR-6792-5p.

### TNFα stimulation of brain and lung endothelial cells

To compare effects of other stimuli with those of iRBCs stimuli, we examined the responses of brain and lung ECs to TNFα stimulation. TNFα stimulation upregulated various immune pathways, such as cytokine signaling pathways (IL10, IL7, and TNF receptor pathways). The Toll-like receptor cascades were also activated in both cells. These results align with other studies that characterized the TNFα effect on ECs.^53^

### Brain EC-specific alterations in miRNA/mRNA profiles in response to infectious stimuli

The present study investigated the response of host ECs to ring-stage iRBCs, which circulate freely within the blood circulation before cytoadhesion. Recent studies have identified significant innate immune functions of ECs, including cytokine secretion, phagocytosis, and antigen presentation.^54^ ECs can also detect pathogen-associated molecular patterns and damage-associated molecular patterns (DAMPs) and elicit proinflammatory immune-enhancing responses and anti-inflammatory immunosuppressive responses.^34^ Previous research demonstrated that trophozoites can activate the endothelium of the BBB (Blood brain barrier) and increase ICAM-1 expression on the EC surface.^55^ There has been some controversy over whether ring stage parasites can stimulate ECs.^55^

Exposure to parasite-infected RBCs altered expression of eight miRNAs in brain ECs, including miR-4509, -142-5p,-144-3p, -342-3p, -497-5p, -103b, -4485-3p, and 4289. A prior study analyzed EV miRNA profiles in EVs mice infected with *Plasmodium ANKA* and *P. yoelii*. The study identified high miR-146a and miR-193b levels in EVs from CM-infected mice compared with EVs from non-cerebral malaria-infected mice and non-infected mice.^56^ Additional studies demonstrated altered expression of 12 miRNAs, including miR-21-5p,-18a-5p,-19a-3p, −20b-5p, -142-3p, -27a-5p, -152-3p, -193a-5p, -155-5p,-218-1-3p, -543, and -411-5p, in mice with CM compared with mice with non-CM. These miRNAs are significantly involved in some CM-like adherens junctions and the FoxO, TGF-β, and endocytosis pathways.^56^ A prior study of a human patient demonstrated that whole blood miR-150-5p was downregulated in an adult infected with *P. vivax*.^57^ Contrastingly, in the present study, we identified that a miR-150-5p isomiR was significantly upregulated in brain ECs exposed to iRBCs. Also, a prior study identified upregulation of miR-150-5p in plasma-derived EVs from patients infected with *Plasmodium vivax*.^57^ In a previous study of experimental malaria, changes in miRNA expression were reported to be linked to the pathogenesis of CM. For example, in the brain of *Plasmodium berghei*-infected mice, miR-27a, miR-150 and let7i levels were upregulated, compared with those in uninfected mice.^58^

### Activation of the endocytosis pathway in brain ECs exposed to iRBCs

We previously demonstrated that iRBCs secrete miRNA-containing EVs^34^ that are presumably taken up by recipient cells, in which cargo miRNAs suppress gene expression. Activation of the endocytosis pathway identified in the present study could indicate EV uptake in brain ECs. EVs are generally internalized into recipient cells by clathrin-dependent endocytosis, which was also increased in brain ECs exposed to iRBCs.^59^^,^^60^ The endocytic system consists primarily of clathrin-containing endocytic vesicles, early and late endosomes. However, clathrin-independent endocytosis, which is less-characterized, also occurs in ECs. The clathrin-dependent pathway is responsible for transporting transferrin in brain ECs. Clathrin-coated buds are localized to the central zone of ECs, with well-developed necks, in contrast to clathrin-coated dome-shaped invaginations on the PM. ECs likely use both clathrin-dependent and clathrin-independent endocytosis pathways. After budding, clathrin-dependent endocytic vesicles fuse with one another or pre-existing early endosomes, causing the vesicles to split and release their coating. The majority of lipid and protein vesicle components destined for recycling to the PM accumulate in the vacuolar head, while soluble contents are concentrated in vesicular regions due to their larger fractional volume.^61^^,^^62^^,^^63^^,^^64^

### Lung EC-specific alterations in miRNA /mRNA profiles in response to iRBC exposure

Severe malaria is associated with acute lung injury (ALI) and acute respiratory distress syndrome (ARDS). The human host responds to infection by initiating a complex inflammatory response in the lungs. This includes cytokine production, neutrophil activation and macrophage activation. Permeability of the alveolar capillary membrane subsequently increases, causing ventilation perfusion mismatch and edematous lung. These interactions compromise lung function.^65^

To our knowledge, we present for the first time, the acute response of lung ECs to ring-stage iRBCs under physiological shear stress. We identified that co-incubation with those iRBCs prominently increased expression of genes encoding ETC components in lung ECs. A recent study reported that the ETC plays a role in immune cell activation, proliferation, and differentiation.^66^^,^^67^ The ETC consists of multimeric protein complexes I-IV, on the inner mitochondrial membrane. Complex I generates reactive oxygen species (ROS), and recent studies have identified critical roles for this interaction in inflammatory macrophages and T helper 17 cells (TH17). Complex II is the site of reverse electron transport in inflammatory macrophages and regulates fumarate levels, which are linked to epigenetic changes. Complex III also produces ROS that activate hypoxia-inducible factor 1-alpha (HIF-1α) and contribute to regulatory T cell (Treg) function. Complex IV is required for T cell activation, differentiation, and Treg subset development. Complex V is required for TH17 differentiation and is sometimes expressed on the surface of tumor cells, where it is recognized by anti-tumor T and NK cells.^68^ More studies are needed to explain how this pathway initiates the immune response in lung ECs during malaria pathogenesis.

miRNA expression profiling identified that miR126 was significantly upregulated in lung ECs exposed to iRBCs under physiological shear stress conditions. miR126 is a well-established pro-angiogenic, pro-survival, and reparative master regulator of ECs lining the extensive pulmonary and systemic vasculature.^68^ miR126 inhibits the migration, proliferation, and survival of human lung microvascular ECs.^69^^,^^70^ Together, the findings presented here newly reveal tissue-specific acute responses of brain and lung ECs stimulated by iRBCs. These findings lay the groundwork for further studies investigating the EC micromechanics that regulate malaria pathogenesis.

### Limitations of the study

To specify and verify the functions of the miRNAs whose expression was altered by iRBCs and/or shear stress, more *in vitro* functional analyses are required.

## Resource availability

### Lead contact

Further information and requests for resources should be directed to the lead contact, Nahla Galal Metwally (metwally@bnitm.de).

### Materials availability

This study did not produce any unique reagents.

### Data and code availability

RNA-sequencing data have been deposited to NCBI and will be publicly available as of the date of publication. The bioproject number is PRJNA1066103. All other original data reported in the paper will be made available by the lead contact upon reasonable request. This paper does not report the original code. Any additional information required to re-interrogate the data reported in this paper is available from the lead contact upon request.

## Acknowledgments

We would like to thank Prof. Egbert Tannich (BNITM) for substantial support at the beginning of the project. We would also like to express special thanks to Dr. Daniel Cadar and Heike Baum (BNITM) for support with NGS sequencing. We also thank Mohsin Shafiq (Institute for Neuropathology, Medical center Hamburg-Eppendorf-Germany) for help and support with Nanosight measurements. We extend special thanks to Monika Rottstegge for the help with the IPA license.

This work was supported by Leibniz Center Infection, 10.13039/100010127Jürgen Manchot Stiftung, German center for infection research and German research foundation (BR 1744/20-1).

## Author contributions

Conceptualization, N.G.M.; methodology, M.d.P.M.T., H.T., J.A., S.M., M.B., P.B, Y.W., T.S., K.H., and N.G.M; software, N.G.M.; validation, K.H., B.H., and N.G.M.; formal analysis, N.G.M.; writing – original draft preparation, N.G.M.; review and editing, M.d.P.M.T., H.T., B.H., H.H., I.B., and N.G.M. All authors have read and agreed to the published version of the manuscript.

## Declaration of interests

The authors declare no competing interests.

## STAR★Methods

### Key resources table

**Table:** 

REAGENT or RESOURCE	SOURCE	IDENTIFIER
**Antibodies**		

Alexa Fluor 488	Thermo Fisher Scientific	#A28175
α human CD29 (integrin beta1)	Biolegend, San Diego, USA	–

**Chemicals**		

RPMI 1640	Applichem	A1538,9010
Hypoxanthine	Sigma	H9636-56
Gelatin from porcine skin-175 g bloom type A	Sigma	G2625
Complete human endothelial cell kit	Cell Biologics®	#H1168
Microvascular endothelial cell growth medium	Provitro®	#2010102

**Experimental models: Cell lines**		

HBMEC (brain ECs)	Cell Biologics	# H-6023
HMVEC-L (lung ECs)	Provitro®	#1210144
Human Serum	Human Serum (A^+^) Interstate Blood Bank, Inc (Memphis, USA).	–

**Experimental models: Organisms/strains**		

IT4 (FCR3S1.2)	In August 1976, 1 mL of patient blood (MRCL, Fajara-Gambia, West Africa) was diluted in 10 mL RPMI 1640 and 10% FCS and shipped on wet ice to New York, NY, USA. Parasites were cultured directly in continuous cultures using the Petri-dish-candle jar technique (Jensen und Trager, 1978), resulting in cell lines FCR3 or IT4. Although the cells were suspected to be mixed with another Brazilian isolate, the cells only contained one genome. This isolate was supplied by Mo Klinkert, BNITM, Hamburg.	–

**Software and algorithms**		

NanoSight Software	(NTA3 0064)	–
Agilent 2100® bioanalyzer software	–	Santa Clara, USA
CLC genomics work bench	version 21	Qiagen, Aarhus
IPA	–	Qiagen, Aarhus
Graphpad Prism	Version 9.4.1	San Diego, California

**Kits**		

QUbit ^TM^ Protein Assay Kit	ThermoFisher Scientific, Waltham, USA	#Q33211
Ultrafiltration units 100,000 MWCO PES	Sartorius, Göttingen, Germany	
miRNeasy mini-Kit	Qiagen, Hilden, Germany	
Agilent 2100® bioanalyzer Pico-Kit	Agilent	
QIAseq Standard mRNA Select Kit (96)	Qiagen	#180775
NextSeq 500/550 Mid Output Kit v.25 (150 Cycles)	Illumina	#20024904

**Data deposit**		

NCBI	PRJNA1066103	

### Experimental model and study participant details

Cellular miRNA and mRNA sequencing were performed after exposing the ECs to multiple stimuli: a) shear stress of 1.5 dyne/cm^2^ (ECs^1.5^), b) shear stress plus fever (40°C) (ECs^1.5+40^°C), c) shear stress plus uninfected RBCs (ECs^1.5+RBCs^), and d) shear stress plus ring stage-iRBCs (ECs^1.5+Rings^). The supernatant of static culture was also collected for EV purification and subsequent miRNA isolation and sequencing. Deep NGS sequencing was then conducted, and data were extracted (20 million reads for miRNA and 6–10 million reads for mRNA). The obtained data were analyzed by CLC genomics, IPA, KEGG, and Reactome analyses. Raw data are uploaded to the NCBI platform (PRJNA1066103).

### Method details

#### Endothelial cell culture

Primary ECs: HBMEC (brain ECs) (Cell Biologics- # H-6023) cultivated in complete human EC kit (Cell Biologics- #H1168) according to Cell Biologics guidelines. HMVEC-L (lung ECs) (Provitro- #1210144) cultivated in microvascular EC growth medium (Provitro- #2010102) according to Provitro guidelines.

#### *Plasmodium* culture

*P. falciparum* isolate IT4/FCR3S1.2 (long-term laboratory adapted), cultivated in RPMI medium with 10% human serum (A+) and 5% hematocrit (0+). The culture was incubated at 37°C in 5% CO2, 1% O2, and 94% N2. Medium was changed daily, and the culture was split according to the experiments’ requirements.^71^^,^^72^ Gelatin flotation was used two days before the flow experiments to isolate *P. falciparum* knobby-trophozoite-iRBCs.^73^ The gelatin enriched trophozoites were taken back to culture and non-infected RBCs were added (5x the amount of trophozoites). On the day of assay, the ring-stage iRBCs were resuspended in 1 mL serum-free RMPI medium to estimate the cell count (1x10^7^ rings were added).

#### Microfluidic pump and shear stress application

As we published previously,^10^^,^^73^ the following steps were performed. A total of 2x10^5^ ECs in one laminar flow slide were seeded 2 days before the assay. ECs were left to adhere for about 2 h. Then 14 mL of ECs culture medium was added into the fluidic unit. One day before the assay, the perfusion set was connected and then air bubbles were removed from the system. The flow was then started with shear stress 1.5 dyne/cm^2^. The fluidic unit and slides were then incubated at 37°C for at least 24 h. On the day of the assay, the medium was then changed to serum-free RPMI medium. Then a total of 1 x 10^7^ highly synchronized iRBCs were transferred to the fluidic unit (715 iRBCs/ul). As a control, RBCS was used in a separate fluidic unit. After the 8 h co-incubation the experiment was stopped. The ECs were then washed with PBS (phosphate buffer solution) and add detached with 200 μL Accutase. The ECs were then collected using serum-free medium and then 500μL TRIzol, was added to the pellet and the pellet was kept at 80°C until further isolation of RNA/miRNA.

#### Extracellular vesicles purification

ECs-EVs were isolated from cell culture with as previously described.^35^^,^^74^ In brief, the medium was changed to vesicle depleted ECs medium (depletion was done by centrifugation at 100,000 x g for 18 h at 4°C). On the next day, (24 h after changing the medium), the cell culture supernatants (15 mL/T75 flask) were collected and sequentially centrifuged at 600 x g, 1600 x g, 3600 x g and 10,000 x g for 15 min each. After each step, the respective supernatant was collected for the next centrifugation step. To concentrate the EVs, the suspension was passed through ultrafiltration units (100,000 MWCO PES; Sartorius, Göttingen, Germany) for 30 min at 3000 x g. The EVs contained in the concentrated supernatant were dissolved in PBS, layered on top of a 60% sucrose cushion, and centrifuged at 100,000 g for 16 h at 4°C. The interphase was collected and washed with PBS twice at 100,000 x g for 60 min at 4°C. EVs were resuspended and pooled in 1000 μL PBS (0.2 μm filtered) and stored in 200 μL aliquots at −80°C. Protein concentration of EV samples was determined using the QUBIT Protein Assay Kit (ThermoFisher Scientific, Waltham, USA) according to manufacturer’s instructions and by measuring the A_280_ content on a Nanodrop2000 (ThermoFisher Scientific, Waltham, USA).

#### mRNA purification and sequencing

Samples in Trizol were thawed before adding 200 μL chloroform and centrifugated for 30 min at 4°C and at 800 x g. The miRNeasy mini-Kit- (Qiagen, Hilden, Germany) was used according to the manufacturer’s instructions. The quality of mRNA/miRNA was assessed using the Agilent 2100 bioanalyzer system. According to the manufacturer’s instructions, Ribosomal RNA was removed using QIAseq FastSelect RNA Removal Kit. The QIAseq Stranded mRNA Select Kit was used for mRNA enrichment. mRNA was sequenced using NextSeq 500/550 Mid Output Kit v2.5 (150 Cycles).

#### The transfer of human microRNA purification and sequencing

Samples in Trizol were thawed before adding 140 μL chloroform and centrifugation for 15 min at 4°C and 800 x g. The miRNeasy mini-Kit- (Qiagen, Hilden, Germany) was used according to the manufacturer’s instructions. The quality of mRNA/miRNA was assessed using the Agilent 2100 bioanalyzer system. MiRNA library preparation was performed in BGI Genomics - China. The small RNAs (18–30 nucleotides) were purified by PAGE. For adapter ligation, the purified RNA was incubated with 3′ adapter followed by the 5′ adapter. Reverse transcription PCR was then performed, and the PCR product was purified by PAGE. After denaturation and circularizing the DNA product, single-stranded circular DNA molecules were replicated via rolling cycle amplification, and a DNA nanoball (DNB) containing multiple copies of DNA was generated. DNBseq-UMI was then performed and about 18 M reads were generated per sample. UMI is known to correct the quantitative bias caused by PCR amplification of more than 70% small RNAs.

#### Electron microscopy

Glow-discharged carbon- and formvar-coated nickel grids (Plano GmbH, Wetzlar, Germany) were incubated with aliquots of freshly isolated EVs. After washing with PBS and incubation in the blocking buffer (0.5% BSA in PBS), the EVs on the grids were labeled with the primary antibody (α human CD29 (integrin beta1), Biolegend, San Diego, USA) at a concentration of 1:100 v/v in PBS (PAA-Laboratories GmbH, Pasching, Austria) containing 0.5% BSA (Sigma-Aldrich, Steinheim, Germany) for at least 21 h at 4°C. The controls for antibody specificity included omitting the primary antibody from the incubating solution. After the incubation period, the grids were rinsed in buffer and further incubated with a goat-*anti*-mouse colloidal gold-conjugated secondary antibody (12 nm gold particles from Jackson Immuno Research, Cambridgeshire, UK) at a dilution of 1:100 v/v for at least 21 h at 4°C. Nickel grids were rinsed in buffer and stained with 2% aqueous uranyl acetate (Electron Microscopy Sciences, Hatfield, USA) for 15 s. Grids were finally observed under a Tecnai Spirit electron microscope (Thermo Fisher Scientific, Waltham, USA) operating at 80 kV, and images were recorded with a digital CCD camera.

#### Nanoparticle tracking analysis (NTA) with Nanosight LM10

The pellet of purified EVs was suspended in PBS 1:300. The following settings were set according to the manufacturer’s software manual (NanoSight LM10 User Manual, MAN0510-04-EN, 2015): the camera level was increased until all particles were distinctly visible (level16 and gain = 20). A total number of 900 frames was recorded in each session (camera: CCD). The autofocus was adjusted to avoid indistinct particles. For each measurement, five 1-min videos were captured under the following conditions: cell temperature: 25°C; Frame rate/FBS: 30. After capture, the videos were analyzed by the in-build NanoSight Software (NTA3 0064) with a detection threshold of 6 and screen gain of 10.

#### Immunofluorescence assays

ECs were seeded on glass slides. The slides with ECs monolayers were fixed in acetone for 30 min and then rehydrated with 1x PBS for 5 min. The first antibody (1:50 in 3% BSA/PBS; α human CD29 (mouse monoclonal), CBL-162, MM2-57, Millipore) was then added and the slides were incubated for 1 h in a humid dark box. The smears were washed 5x with 1X PBS and then labeled with AlexaFluor (1:1000) (Thermo Fisher # A28175) and DAPI (1 mg/mL) (1:1000) (Roche # 10236276001) for 1 h in a humid dark box. After a 5x wash with 50 μL 1x PBS, the smears were air dried and Moviol was added before they were covered with a plastic coverslip. Images were taken through a EVOS FL auto-inverted microscope (Thermo Fisher Scientific, Waltham, USA) and analyzed using ImageJ 1.53K. To calculate the corrected total cell fluorescence, the following formula was used: Integrated Density – (Area of selected cell X Mean fluorescence of background readings).

### Quantification and statistical analysis

Bioinformatics analysis was conducted using CLC genomics work bench version 21 (Qiagen, Aarhus). Clean reads were imported, and miRNA was quantified and annotated on the miRbase v22. Differential expression was performed, and P-values were adjusted using FDR 10%. miRNAs with extremely low abundance were excluded from our analysis. Hsg38 was used as a mRNA reference.

#### CLC Parameters (miRNA-Quantification)

miRBase: miRBase-Release_v22/Prioritized species = Homo sapiens/Allow length based isomiRs = Yes/Additional upstream bases = 2/Maximum mismatches = 2/Strand specific = Yes/Minimum sequence length = 18/Maximum sequence length = 25.

#### CLC differential expression parameters (the transfer of human microRNA/mRNA)

Whole transcriptome RNA-seq/Normalization Method = TMM/Filter on Average expression for FDR correction/Result Handling = Save.

#### CLC trim parameters (mRNA)

Trim using quality scores = Yes/Quality limit = 0.05/Trim ambiguous nucleotides = Yes/Maximum number of ambiguities = 2/Automatic readthrough adapter trimming = Yes/Remove 5′ terminal nucleotides = No/Remove 3' terminal nucleotides = No/Trim to a fixed length = No/Maximum length = 150/Trim end = Trim from 3′ end/Discard short reads = No/Discard long reads = No/Save discarded sequences = No/Save broken pairs = No/Create report = Yes.

#### CLC RNA-seq parameters (mRNA)

Enable spike-ins = No/Database files = Homo sapiens (hg38) sequence/Maximum cost = 2/Similarity fraction = 0.8/Auto-detect paired distances = Yes/Maximum number of hits for a read = 10/Strand setting = Both/Minimum supporting count = 5/Create report = Yes/Unmapped reads = No/Expression value = Total count.

#### Ingenuity Pathway Analysis the transfer of human microRNA target identification parameters

QIAGEN IPA Software, copyright 2023, was utilized for miRNA target filtration and canonical pathway analysis. This software is web-based and gathers information from publicly available databases containing published relationships, mechanisms, biological functions, canonical pathways, and networks. IPA predicts miRNA regulation of target mRNAs based on data from miRBase, TargetScan, and the QIAGEN Knowledge Base. The filters were based on high-confidence predicted and experimentally validated data only. The included canonical pathways were cellular immune responses, cytokine signaling, and pathogen.

## Supplemental information

Document S1. Figures S1–S6

Table S1. miRNA profiles of HBMECstatic and HMVEC-Lstatic

Table S2. mRNA profiles of HBMECstatic and HMVEC-Lstatic

Table S3. miRNA profiles of HBMEC-EVs and HMVEC-L-EVs without and with TNF stimulation

Table S4. miRNA profiles of HBMEC1.5 and HBMECstatic

Table S5. mRNA profiles of HBMEC1.5 and HBMECstatic

Table S6. miRNA-mRNA targets within HBMEC1.5

Table S7. miRNA profiles of HBMEC1.5+Rings and HBMEC1.5+RBCs/ miRNA-mRNA targets within HBMEC1.5+rings

Table S8. miRNA profiles of HMVEC-L1.5 and HMVEC-Lstatic/ mRNA profiles of HMVEC-L1.5 and HMVEC-Lstatic

Table S9. miRNA profiles of HMVEC-L1.5+RBCs and HMVEC-L1.5+Rings/ mRNA profiles of HMVEC-L1.5+RBCs and HMVEC-L1.5+Rings

Table S10. miRNA-mRNA targets within HMVEC-L1.5+Rings

Table S11. miRNA profiles of HBMEC1.5 and HBMEC1.5 + 40°C/ mRNA profiles of HBMEC1.5 and HBMEC1.5 + 40°C/ miRNA-mRNA targets within HBMEC1.5 + 40°C

Table S12. miRNA profiles of HMVEC-L 1.5 and HMVEC-L 1.5 + 40°C/ mRNA profiles of HMVEC-L 1.5 and HMVEC-L 1.5 + 40°C
