# Supplementary material for: Distinct brain and lung endothelial miRNA/mRNA profiles after exposure to Plasmodium falciparum-infected red blood cells
Source: iScience. 2024 Oct 28;27(11):111265. doi: 10.1016/j.isci.2024.111265 (PMC11576406; doi:10.1016/j.isci.2024.111265)
Supplement: Document S1. Figures S1–S6 [file mmc1.pdf]

iScience, Volume *27*

Supplemental information

Distinct brain and lung endothelial miRNA/mRNA profiles after exposure to *Plasmodium falciparum*-infected red blood cells

Nahla Galal Metwally, Maria del Pilar Martinez Tauler, Hanifeh Torabi, Johannes Allweier, Sara Mohamed, Maryeva Bessemoulin, Philip Bouws, Fatima Alshikh, Yifan Wu, Milad Temori, Tabea Schell, Maximillian Rakotonirinalalao, Barbara Honecker, Katharina Höhn, Thomas Jacobs, Holger Heine, and Iris Bruchhaus

Supplementary Figure1

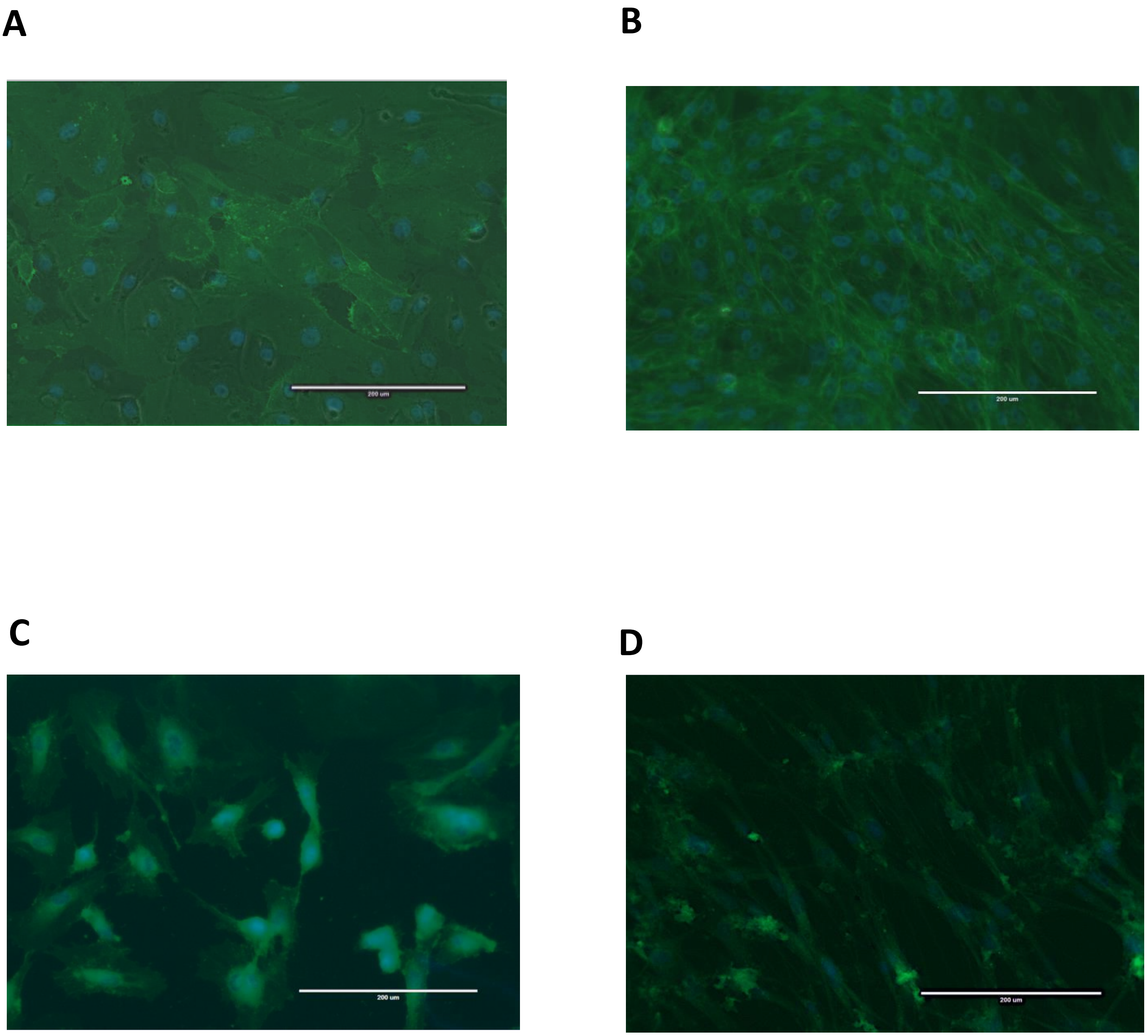

Supplement Figure 1: Immunofluorescence assays of ECs. (A) Immunofluorescence staining of HBMEC^{static} with EC marker CD29. **(B)** Immunofluorescence staining of HMVEC-L^{static} with EC marker CD29. **(C)** Immunofluorescence staining of HBMEC^{static} with EC marker PECAM. **(D)** Immunofluorescence staining of HMVEC-L^{static} with EC marker PECAM. The photos were taken by 20X – EVOS FL auto-inverted microscope.

Supplementary Figure2

A

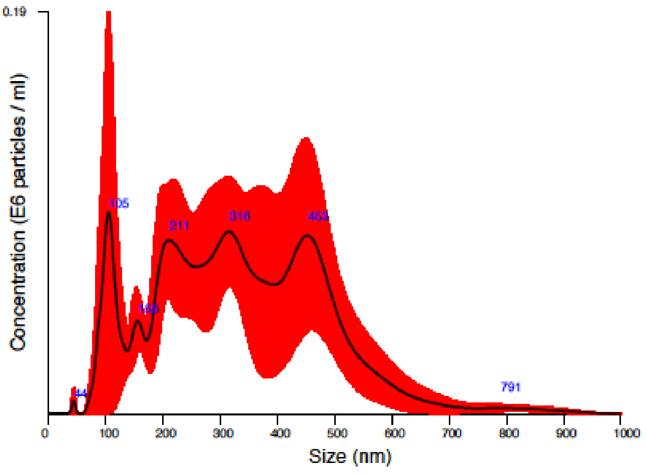

B

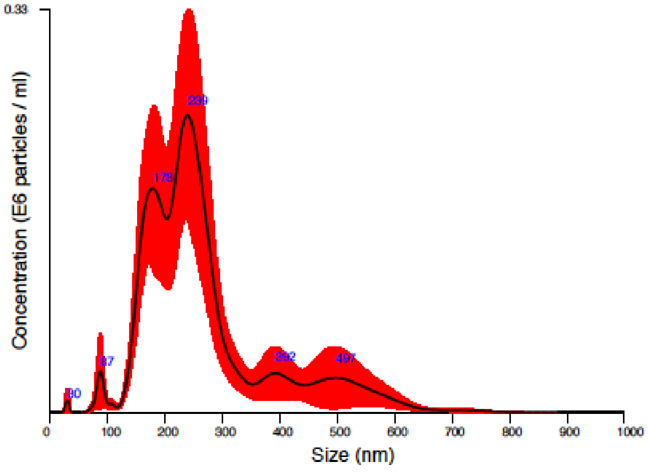

Supplement Figure 2: Nanoparticle tracking analyses of EVs from (A) HBMEC^{static} **and (B)** HMVEC-L^{static}**.**

Supplementary Figure3

A

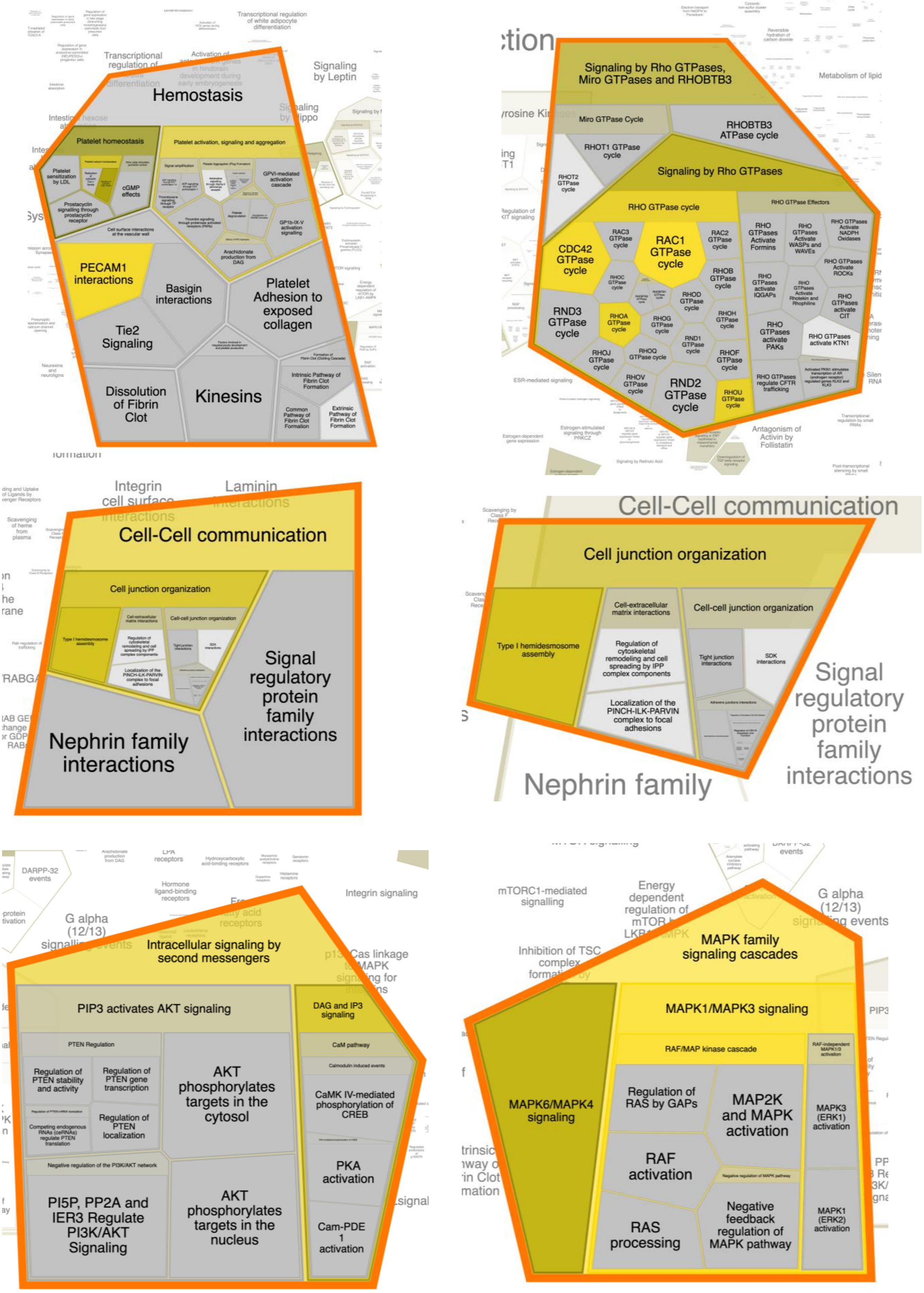

Supplement Figure 3: Reactome pathways that were significantly regulated in HBMEC^{static}

Supplementary Figure 4

B

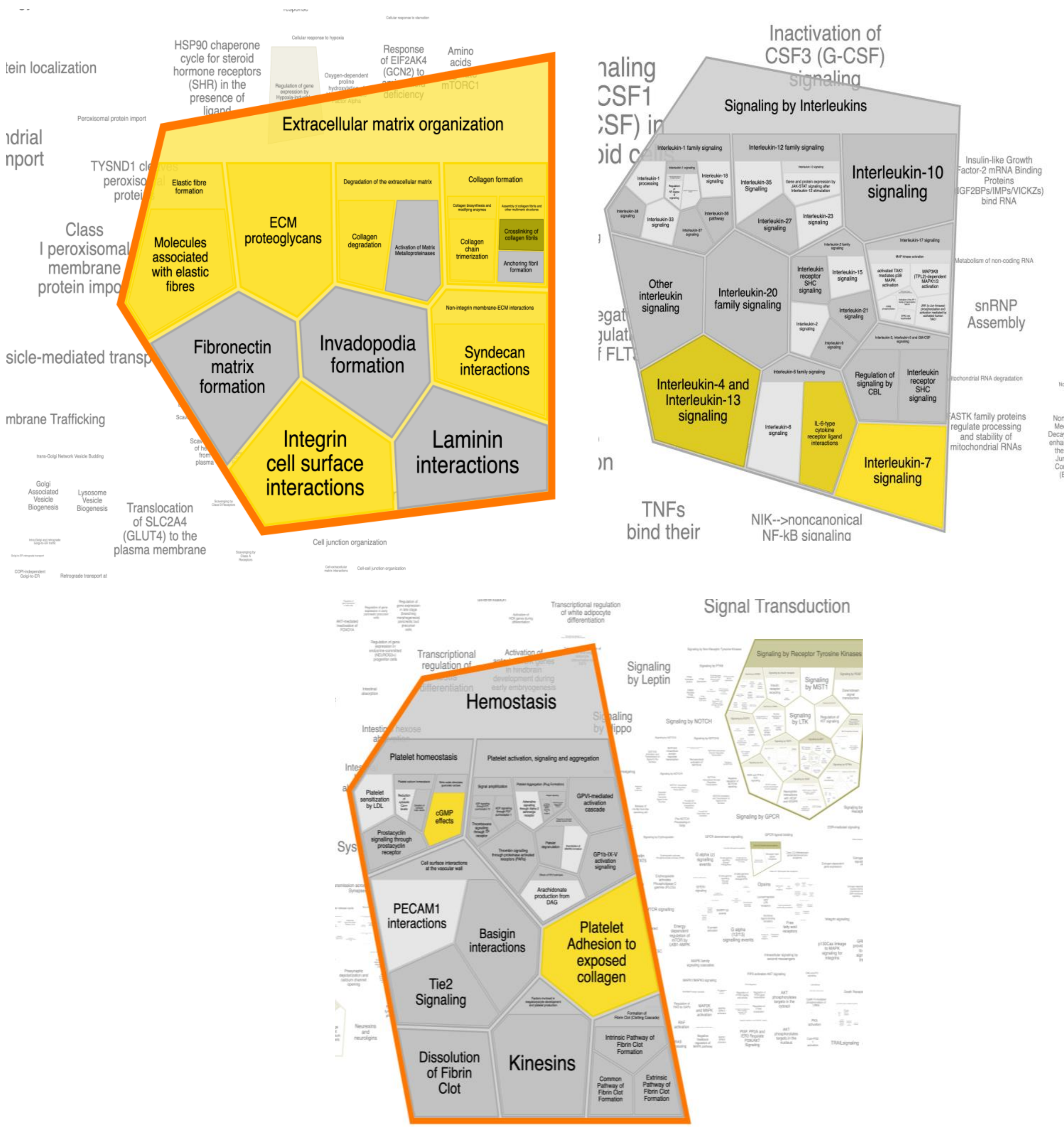

Supplement Figure 4: Reactome pathways that were significantly regulated in HMVEC-L^{static}.

Supplementary Figure 5

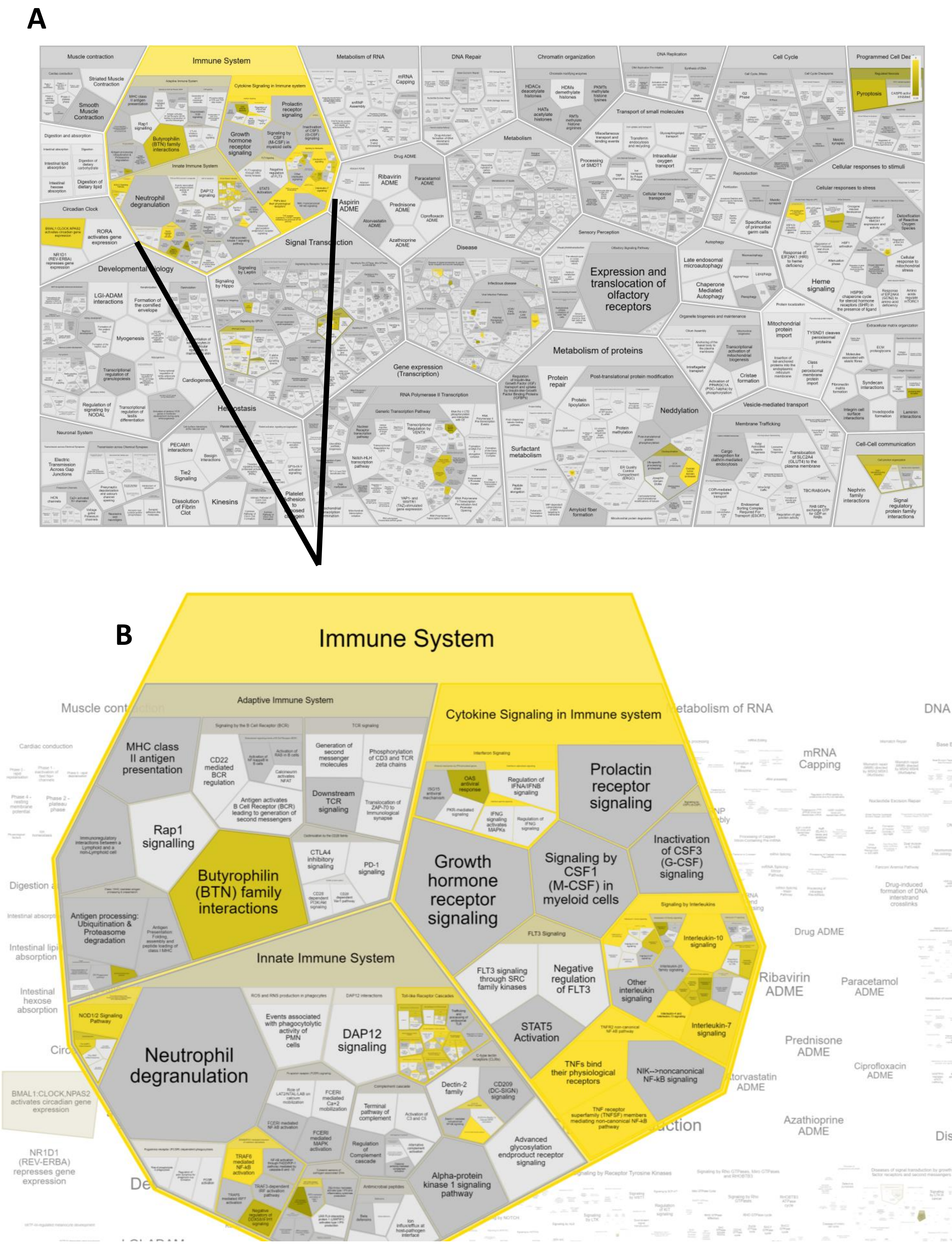

Supplement Figure 5: Reactome pathways that were significantly regulated in HBMEC$^{static+TNF\alpha}$

Supplementary Figure 6

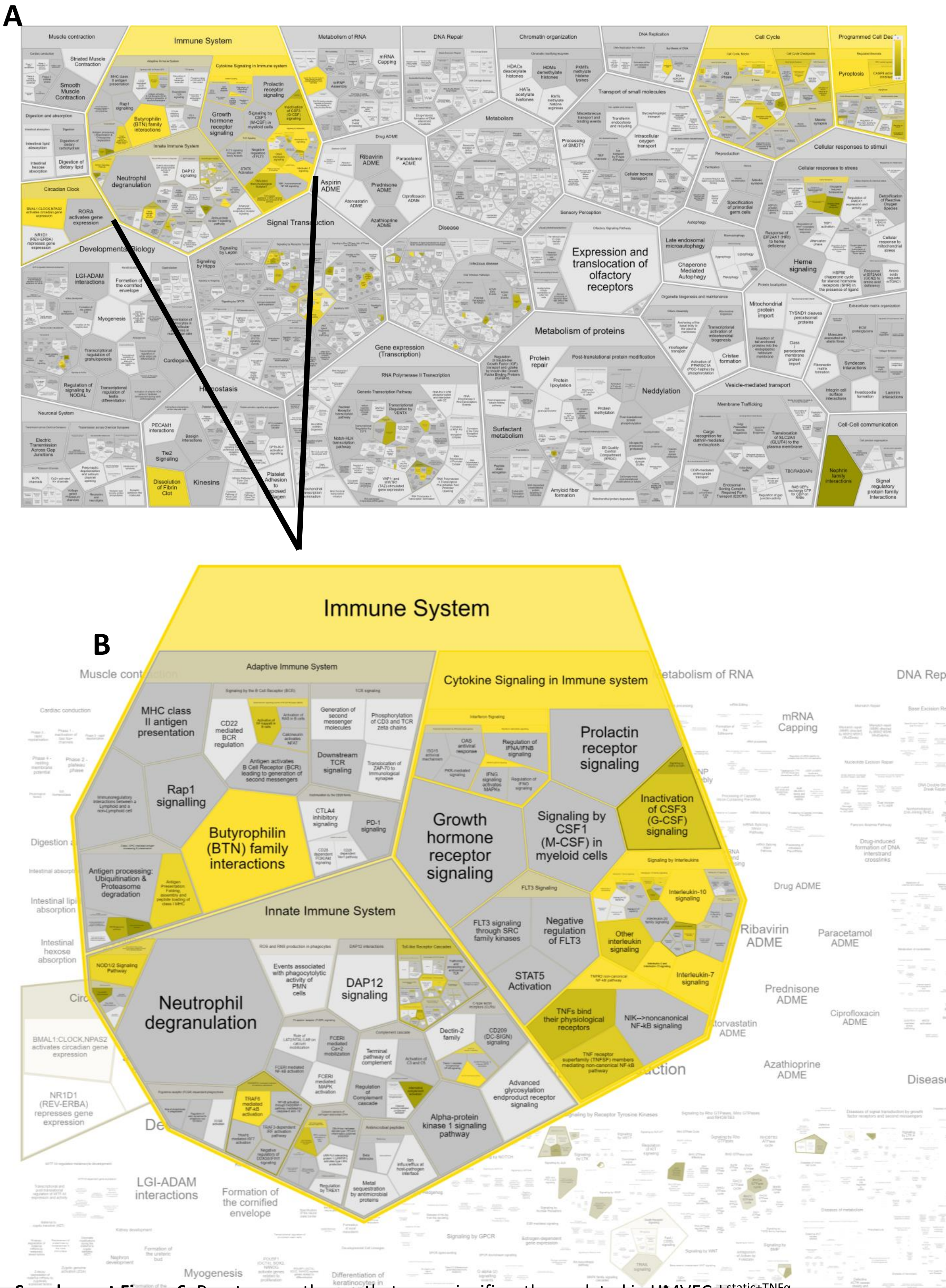

Supplement Figure 6: Reactome pathways that were significantly regulated in HMVEC-L$^{static+TNF\alpha}$
